# L-Ascorbic Acid-g-Polyaniline Mesoporous Silica Nanocomposite for Efficient Removal of Crystal Violet: A Batch and Fixed Bed Breakthrough Studies

**DOI:** 10.3390/nano10122402

**Published:** 2020-11-30

**Authors:** Imran Hasan, Ibtisam I. BinSharfan, Rais Ahmad Khan, Ali Alsalme

**Affiliations:** 1 Environmental Research Laboratory, Department of Chemistry, Chandigarh University, Gharuan, Mohali, Punjab 140301, India; imranhasan98@gmail.com; 2 Department of Chemistry, College of Science, King Saud University, Riyadh 11451, Saudi Arabia; 437202977@student.ksu.edu.sa (I.I.B.); krais@ksu.edu.sa (R.A.K.)

**Keywords:** mesoporous silica NPs, fixed bed adsorption, Box–Behnken design, mass transfer coefficient, Thomas model

## Abstract

In the present study, mesoporous silica nanoparticles (MSNs) synthesized through sol–gel process and calcined at 600 °C were further surface functionalized by a copolymer chain of L-ascorbic acid (AS) and polyaniline (PAni) by in situ free radical oxidative polymerization reaction. The surface modification of MSNs by AS-g-PAni was confirmed by using various analytical techniques, namely FTIR, XRD, SEM–EDX, TEM and AFM. The composition of AS-g-PAni@MS was found to be composed of C (52.53%), N (20.30%), O (25.69%) and Si (1.49%), with 26.42 nm as the particle size. Further, it was applied for the adsorption of crystal violet (CV) dye under batch, as well as fixed bed method. RSM–BBD was taken into consideration, to optimize the various operational parameters effecting the adsorption through batch method. To explore maximum efficiency of the material, it was further subjected to adsorption of CV under fixed bed method, using the variable bed heights of 3.7, 5.4 and 8.1 cm. Based on high value of regression coefficient (R^2^) and low value of RMSE given as (0.99, 0.02) for 3.7 cm, (0.99, 0.03), the breakthrough data were very well defined by the Thomas model, with optimum concurrence of stoichiometric adsorption capacity values. The external mass transfer equilibrium data were well fitted by the Langmuir model, with maximum monolayer adsorption capacity of 88.42 mg g^−1^ at 303 K, 92.51 mg g^−1^ at 313 K, 107.41 mg g^−1^ at 313 K and 113.25 mg g^−1^ at 333 K. The uptake of CV by AS-g-PAni@MS was well defined by pseudo second order model with rate constant K_2_ = 0.003 L mg^–1^ min^–1^ for 50 and 0.003 L mg^–1^ min^–1^ for 60 mg L^–1^ CV. The adsorption reaction was endothermic with enthalpy (ΔH) value of 3.62 KJ mol^−1^ and highly efficient for treatment of CV-contaminated water for more the five consecutive cycles.

## 1. Introduction

The thriving development in the field of nanotechnology over the past two decades has delivered apt approached to amortize the environmental hazard in aquatic system pertaining to advanced properties of nanomaterials like morphological, rheological, adhesive, molecular, mechanical and sensing [1,2,3,4,5]. Recently, mesoporous silica nanoparticles (MSNs) and their nanocomposite materials have attracted the researcher’s attention in many fields like extraction, drug delivery, catalysis, optoelectronics and adsorption [6,7,8,9]. With a consideration of high porosity and aspect ratio, MSNs have been widely applied with prominent interest in adsorption technology for scavenging various types of inorganic and organic hazards from aquatic system [10,11]. In the literature various type of sol–gel process utilized surface functionalized mesoporous silica nanoparticles like CEL-PGA-MSN [12], RGD peptide–MSN [13], Dy_x_MnFe_2−x_O_4_–MSN [14], Fe@Al-MSN [15], MMSNPs [16], TA-MSN-NH_2_ [17], etc., have been used for adsorption of various dyes, inorganic ions and biomolecules. Based on the testimonials augmented in various publications and patens, it was observed that MSNs can be of the standard to fulfil the insatiable society’s demand for the novel and applicable advanced materials [18,19].

By taking the advantage of their chemical inertness and biocompatible properties, they have been utilized in diverse scavenging process with a variation of surface functionalization [11]. Here in the present study the MSNs are surface functionalized by a copolymer blend of natural biomolecule L-ascorbic acid (vitamin C) and synthetic conducting polymer polyaniline (PAni). L-ascorbic acid is a naturally occurring water-soluble organic acid which is present in various foodstuffs and biological systems [20]. The chemical formula of ascorbic acid is 2-oxo-L-thero-hexono-1,4-lactone-2,3-enediol, which is Ɣ- lactone structure with L- enantiomer with both reducing agent and stabilizing properties [21]. Ascorbic acid was found to be highly unstable when exposed to light and converts to dehydroascorbic acid by releasing hydroxyl radicals, so it was derivatized by PAni for inducing more stability in the chemical structure with enhanced adsorptive effects [22]. Polyaniline (PAni) similar to polypyrrole and polythiophene also has been recognized as a conducting polymer enriched with reactive –NH– groups in polymer chains [23]. PAni with tunable conductivity between those of conducting and non-conducting materials, environmental constancy, pH sensitivity (acid/base doping response) and stability at ambient conditions with low cost has made its place among the material of great interest in adsorption technology [24,25].

Among the various inorganic and organic hazardous pollutant discharges by various industrial and agriculture effluents, crystal violet is the key pollutant which is of great concern to both human as well as aquatic life because of its toxic, carcinogenic and non-biodegradable nature [26,27]. Crystal violet (CV) is a cation triphenylmethane class dye which is generally used as textile colorant and biological stain [28]. Furthermore, being a recalcitrant molecule crystal violet (CV) is highly water-soluble CV and even presence of 1 mg L^−1^ (1 ppm) can produce inhibitive characters on the photosynthesis of aquatic plants [29]. Thus, based on the data obtained through various health organizations and water-conserving bodies, CV was categorized as a pollutant of a great concern due to its effect on mammalian cells causing skin allergies, digestive tract irritation and kidney failure [30,31]. These characteristics made the researchers around the globe formulate effective methods to decolorize CV-dye-laden water or remove it from aquatic environments. However, there are various physicochemical and biological methods that have been defined in the literature which have been proved to be best to treat CV polluted water [32,33]. Among these methods, adsorption technology was selected as suitable and efficient based on the material structure and its chemical reactivity to treat CV-laden effluents [34,35].

The response surface methodology (RSM) is recognized worldwide as the best statistical and mathematical tool for optimizing reaction parameters with good precession and high desirability values [36]. Under the RSM, the subcategory is the Box–Behnken design (BBD), which optimizes the number of experiments to be carried out to ascertain the possible interactions between the parameters studied and their effects on the removal of CV [37,38]. The goal of the current study was to find the most efficient method for the removal of CV in wastewater by using synthesized AS-g-PAni@MS nanocomposites. The efficiency of the synthesized material was also explored by using fixed bed breakthrough studies by varying the bed heights to 3.7, 5.4 and 8.1 cm.

## 2. Materials and Methods

### 2.1. Chemicals

We used tetraethyl orthosilicate (TEOS d = 0.934 g/mL 99%, Aldrich, Bangalore, India), Cetyltrimethylammonium ammonium bromide (CTAB 98%, Aldrich, Bangalore, India), ethanol (d = 0.789 g/mL ≥ 99.8%, Fluka, New Delhi, India), ammonium hydroxide (d = 0.90 g/mL 30%, Loba Chemie, Mumbai, India) and hydrochloric acid (37%, Aldrich, Bangalore, India). Crystal violet (>88%, d = 1.19 g cm^−3^) was supplied by Merck Millipore, India, having maximum absorbance in the range of λ_max_= 584–590 nm. L-ascorbic acid (>99%) was purchased from Sigma Aldrich, Bangalore, India, and Aniline (Thermo Fischer Scientific, Mumbai, India) was purified by distillation over zinc dust. The middle fraction of the distillate was collected and stored in a refrigerator. All chemicals were without any further modification, purification or distillation. Deionized water (DI) was used for all experiments.

### 2.2. Synthesis of Mesoporous Silica Nanoparticles (MSNs)

The proposed mesoporous silica nano particles (MSNs) were prepared by hydrolysis and condensation methods, reported elsewhere, with some modification [12]. In a conical flask, a mixture of 100 mL ethanol, 5 mL of TEOS, 2 g CTAB and 20 mL concentrated ammonia (25%) in 150 mL distilled water was taken and left over magnetic stirring (500 rpm), at 25 °C, for 1 h. After the mixture became homogenized, an additional 15 mL of TEOS was added to the mixture, and the reaction was left over stirring for 5 h. In order to stop the base catalyzed reaction, a 10 mL aliquot of 1 M HCl was added drop wise to form the suspension which was left out, standing, for 24 h. The MSNs were collected by centrifuge (Remi Laboratory Instruments, Mumbai, India, 800 rpm) and washed with a mixture of distilled water and ethanol, to remove any unreacted residue of CTAB and NH4Cl formed during the reaction. The MSNs were dried in a hot-air oven, at 80 °C, and later calcined at 600 °C, in a temperature-controlled furnace for 4 h.

### 2.3. Synthesis of AS-g-PAni@MS Nanocomposite

**Figure 1:**
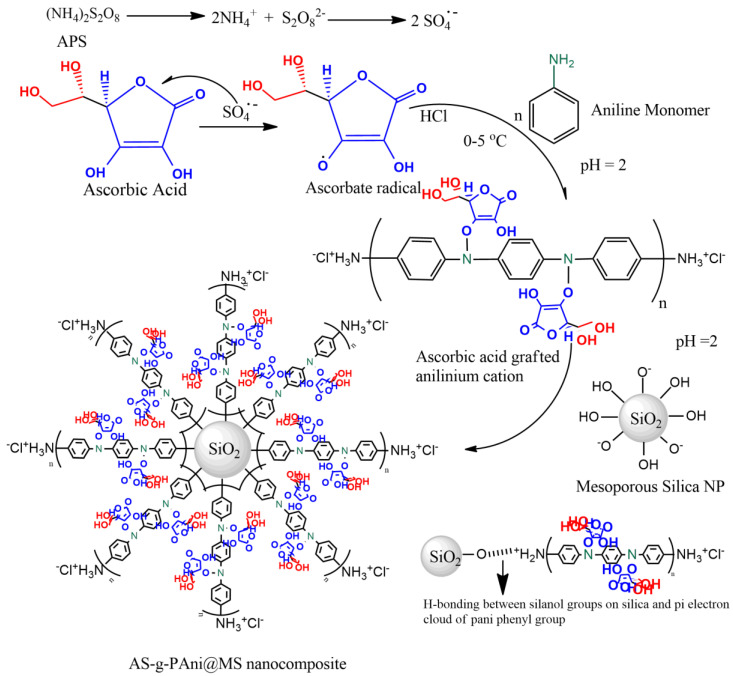
Synthesis scheme for AS-g-PAni@MS nanocomposite material through in situ free radical polymerization reaction.

The synthesis of the material was accomplished by in situ free radical polymerization of aniline monomer in presence of ascorbic acid and MSNs [24]. A dispersion was made by pouring out 1.5 g of MSNs in 100 mL 0.5 M HCl solution under the sonication. To this dispersion, 2 g of ascorbic acid was added, and mixture was left on stirring for 2 h, at room temperature. After complete homogenization, a solution 15 mL of distilled aniline monomer was added in the solution, and again the reaction was left another 2 h. The polymerization was started after the drop by drop addition of 2 mmol ammonium persulphate solution (APS) to the mixture. After adding a 30 mL addition of APS, the reaction was left on stirring for 12 h. The progress of reaction was observed through change in color. Finally, an emerald green suspension was obtained which was filtered and washed several times with deionized water, to remove any unreacted aniline and HCl entities. The material was dried in a hot-air oven for 4 h, at 60 °C, and collected in CaCO_3_ desiccators for further analytical characterization and adsorption experiments. The complete synthesizing scheme of the material is given in Figure 1.

### 2.4. Analytical Techniques Used for Material Characterization

The crystal structure and properties of the product polymer was determined using FTIR, XRD, Transmission electron microscopy (TEM), energy dispersive X-ray (EDX), scanning electron microscopy (SEM) and Atomic Force Microscopy (AFM). The type of bonding interactions and functional groups present in the nanocatalyst was determined via Fourier-transform infrared spectroscopy (FTIR) using a Perkin Elmer PE1600 spectrometer (Ramsey, MN, USA). FTIR analysis was conducted in the 400–4000 cm^−1^ frequency range with the transmission mode. A Rigaku Ultima 1V XRD diffractometer (Tokyo, Japan) was employed to assess the crystalline structure of the nanocomposites. TEM was used to determine the particle size of the nanocomposites. Here, the elemental size and dispensation of the nanocomposite materials in the blended polymer matrix were determined using a JEM 2100 electron microscope (Akishima, Tokyo, Japan). The surface morphological features, elemental identification, and both the chemical composition and homogeneity of AS-g-PAni@MS were determined via SEM combined with EDX–SEM; JEOL GSM 6510LV (Akishima, Tokyo, Japan). A Shimadzu UV-1900 UV–Vis double-beam spectrophotometer (Kyoto, Japan) was utilized to analyse aliquots of the CV samples after completion of the adsorption reaction.

### 2.5. Experiment Design and Adsorption Experiments

The experimental design was subsequently executed via the RSM-coupled BBD to establish the synergistic or antagonistic effects of three or more variables on the efficiency of the nanocomposite material for CV removal [39]. The entire design mainly consisted of four parameters as contact time (A), pH of CV solution (B), CV concentration (C) and adsorbent dose (D). The complete variable with their −1, 0 and +1 range are given in Supplementary Materials Table S1. For the efficient removal of CV by AS-g-PAni@MS, the abovementioned variables can be articulated by using the quadratic regression equation:

(1) $$y=b_{0}+\sum_{i=1}^{n}b_{i}x_{i}+\sum_{i=1}^{n}b_{ii}x_{i}{}^{2}+\sum_{1\leq i<j}^{n}b_{i}{}_{j}x_{i}x_{j}+\varepsilon$$

where *x_i_* and *x_j_* represent the linear function that transforms the original actual values *X_i_–αC_i_*, *X_i_–C_i_*, *X_i_*, *X_i_+C_i_* and *X_i_+αC_i_* to the coded values −α, −1, 0, +1 and α and b_0_ is constant coefficient, the coefficient of first order effect is represented by *b_i_*, *b_ij_* is the coefficient of interaction with *ε* as coefficient of higher terms [40]:

(2) $$x=\frac{\left(X-x_{i}\right)}{C_{i}}$$

The selective scavenging process of CV by AS-g-PAni@MS nanomaterial was done through batch experimental methods. Based on the variable ranges given in Supplementary Materials Table S1, BBD provided a design table given in Supplementary Materials Table S2 consist of 29 random order experiments with different sets of variable values. An aliquot of 20 mL CV was used with each set of Sonication Time A (60–120 min), pH of the medium B (4–7), CV dye concentration C (30–60 mg L^−1^) and adsorbent dose D (20–40 mg) variables in batch mode at 333 K temperature and the residual concentration of CV after the adsorption experiment was assessed by the UV–Vis spectrophotometer as maximum wavelength (λ_max_) of 585 nm. The total removal rate (%) of CV and the adsorption capacity of synthesized material was calculated by Equations (3)–(5):

(3) $$\%\;\mathrm{CV}\;\mathrm{Removal}=\frac{C_{o}-C_{t}}{C_{o}}\times 100$$

(4) $$q_{e}=\frac{(C_{o}-C_{e})\times V}{m_{ads}}$$

(5) $$q_{t}=\frac{(C_{o}-C_{t})\times V}{m_{ads}}$$

where *C_o_* is the initial concentration of *CV* (mg L^−1^), *C_e_*, *C_t_* are the equilibrium and at time t *CV* concentration (mg L^−1^), *q*_e_, *q_t_* are the adsorption capacities of material at equilibrium and at time t (mg g^−1^). *V* is the volume of the *CV* aliquot taken (L) and *m_ads_* the mass of the adsorbent taken during the analysis (g). The analysis was repeated at least thrice to determine the uncertainties values.

### 2.6. Adsorption Isotherms and Thermodynamic Analysis

The isotherm studies were carried to find out the frequency of the adsorption process as how the substrate molecule is going to distribute itself between the liquid phase and solid phase under the influence of residual forces and concentration gradient. In the literature, various adsorption isotherm models have been reported. In our present study, the adsorption data were explored with the Langmuir and Freundlich models followed by linear regression [41,42]. The linear equations are given by Equations (6) and (7):

(6) $$\frac{C_{e}}{q_{e}}=\frac{1}{{}_{\;}q_{m}K_{L}\;\;}+\frac{C_{e}\;}{q_{m}}$$

(7) $$\ln q_{e}=\frac{1}{{}_{\;}n\;\;}\ln C_{e}\;+\ln K_{F}$$

where *q_m_* is the maximum monolayer adsorption capacity (mg g^−1^), *K_L_* the Langmuir constant (L mg^−1^), *K_F_* is the Freundlich constant (mg g^−1^)(L mg^−1^)^1/*n*^ and n is a constant measuring the extent of favorability of adsorption with respect to change in conditions such as *n* > 1 value represents a favorability while *n* < 1 is measure of non-favorability. The efficiency and degree of feasibility of the CV removal by AS-g-PAni@MS nanocomposite was evaluated by thermodynamic studies. Various thermodynamic parameters such as Enthalpy (Δ*H*), Entropy (Δ*S*) and Gibbs free energy (Δ*G*) were used to find out the temperature-based variation of the process. The equations for thermodynamic studies are given by Equations (8)–(10) [43]:

(8) $$K_{c}=\frac{C_{ad}}{C_{e}}$$

(9) $$\ln K_{c}=-\frac{\Delta H_{}^{0}}{R}+\frac{\Delta S_{}^{0}}{RT}$$

(10) $$\Delta G_{}^{0}=\Delta H_{}^{0}-T\Delta S_{}^{0}$$

where *C*_ad_ (mg L^−1^) is the CV concentration adsorbed on the surface of the AS-g-PAni@MS nanocomposite, *K_c_* is the distribution coefficient, *R* is the gas constant (8.314 J K^−1^ mol^−1^) and *T* is the temperature of the system (303–333 K).

### 2.7. Kinetic Models

To analyse the rate controlling step for the sequestration of CV on AS-g-PAni@MS surface kinetic studies were performed using 50 and 60 mg L^−1^ CV in the time range of 5–150 min, using 20 mg (0.02 g) adsorbent at pH 6.9. The data obtained by experimentation were applied to Pseudo First order and Pseudo second order model followed by non-linear regression. The equations for both the models are given by the following [44,45]:

(11) $$q_{t}=q_{e}(1-e^{-k_{1}t})$$

(12) $$q_{t}=\frac{k_{2}q_{e}{}^{2}t}{1+k_{2}q_{e}t}$$

where *q_e_*, *q_t_* are the adsorption capacities of material at equilibrium and at time t (mg g^−1^) while *k*_1_ (min^−1^) and *k*_2_ (g mg^−1^ min^−1^) are related to the rate constants for pseudo first and second-order models.

### 2.8. Fixed Bed Continuous Flow Studies (Breakthrough)

This study was performed to analyse the CV removal capacity of AS-g-PAni@MS by mass transfer method through diffusion process. For this purpose, a borosilicate glass column of 3.0 cm and 25.0 cm length was utilized with 60 mg L^−1^ CV concentration with a flow rate of 10 mL min^−1^ with variable bed heights as 3.7, 5.4 and 8.1 cm. The fixed bed was made by pouring out appropriate amount of the adsorbent between the glass wool and glass beads as supporting layers to prevent loss and clogging of the adsorbent during operation. The flux of the dye solution in the present study was made to flow through the adsorbent bed under gravitational force and atmospheric pressure with descending mode. The pH of the solution was 6.9 (7.0) as obtained through our BBD design experiments. The effluents are collected with respect to time and assessed using a double beam spectrophotometer at 585 nm. Different fixed bed parameters such as total volume of treated effluent (*V_eff_*, L), the equilibrium adsorption capacity (*q_eq_*, mg g^−1^), length of mass transfer zone (*Z_m_*, cm) and CV removal percent was calculated by Equations (13)–(16) [46,47,48,49]:

(13) $$V_{eff}=Q\times t_{total}$$

(14) $$q_{eq}=\frac{QC_{o}}{m_{ads}}\int_{0}^{t_{total}}(1-(\frac{C_{t}}{C_{o}}))dt$$

(15) $$Z_{m}=L(1-\frac{t_{b}}{t_{e}})$$

(16) $$\%R=\frac{100}{t_{total}}\int_{0}^{t_{total}}(1-(\frac{C_{t}}{C_{o}}))dt$$

The obtained data from the fixed bed breakthrough studies were further applied to the Thomas model for verification of data fitting. The equation for the Thomas model is given by Equation (17) [50]:

(17) $$\frac{C_{t}}{C_{o}}={\left(1+\exp(q_{th}k_{th}\frac{m_{ads}}{Q}-k_{th}C_{o}t)\right)}^{-1}$$

where *t_total_* is the total time used to reach equilibrium point, *Q* is the flow rate (mL min^−1^), *m_ads_* mass of the adsorbent (g), *t_b_*, *t_e_* is the breakthrough time and exhaustion time (min), *k_th_* is the Thomas rate constant (mL mg^−1^ min^−1^) and *q_th_* is the stoichiometric Thomas adsorption capacity (mg g^−1^).

### 2.9. Statistical Verification of Data

The combination of statistical error analysis tool with the obtained data was employed to find out the more precise kinetic model that plays a critical role during the photodegradation reaction. So, root mean square error (RMSE) was taken into consideration with regression coefficient to optimize the data values for most preferential model. The equation for RMSE is given by Equation (18) [51]:

(18) $$RMSE=\sqrt{\sum_{i=1}^{n}{\left(q_{e,cal}-q_{e,\exp}\right)}_{i}{}^{2}}$$

## 3. Results and Discussion

### 3.1. Analytical Techniques for Material Characterization

**Figure 2:**
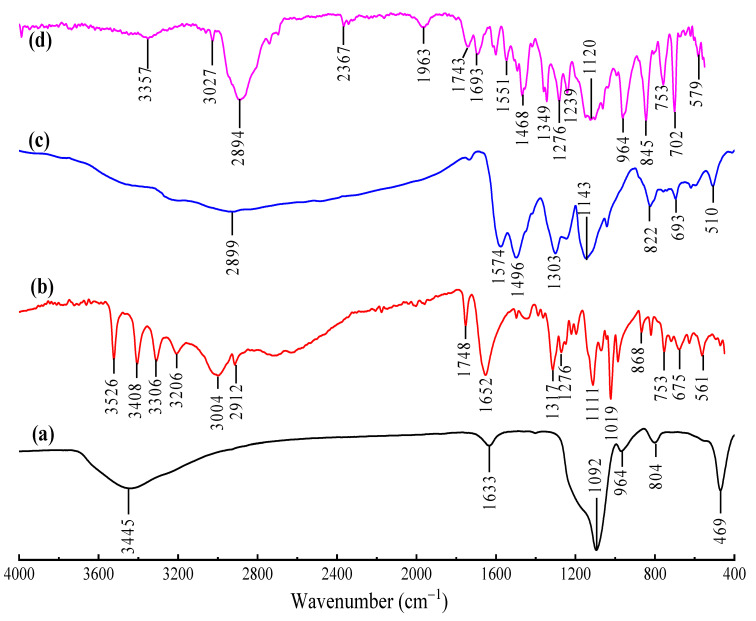
FTIR spectra of (**a**) calcined mesoporous silica nanoparticles (MSNs), (**b**) L-ascorbic acid, (**c**) pure PAni and (**d**) AS-g-PAni@MS nanocomposite.

Figure 2 shows the FTIR spectra of (a) calcined MSNs, (b) L-ascorbic acid, (c) PAni and (d) AS-g-PAni@MS nanocomposite. The FTIR spectra of MSNs represent characteristics peaks at 3445 cm^−1^ (Si–OH stretching vibrations), 1633 cm^−1^ (O–H bending vibrations), 1092 cm^−1^ (Si–O–Si asymmetric stretching vibrations), 964 cm^−1^ (Si–O–H_2_O bending vibrations), 804 cm^−1^ (Si–O bending vibrations) and 469 cm^−1^ (Si–O rocking vibrations) [7]. The FTIR spectra ascorbic acid shows peak at 3207–3790 cm^−1^ (–OH stretching of four different –OH groups), 2733, 3003 cm^−1^ (–CH in aliphatic and ring stretching), 1752 cm^−1^ (–C=O carbonyl stretching), 1659 cm^−1^ (C–O stretching), 1107, 1116 cm^−1^ (C–O–C stretching) and 675, 751 cm^−1^ (–OH out of plane deformation) [21]. The FTIR spectra of PAni show characteristic peaks at 1574, 1496 cm^−1^ (stretching vibration mode of C=C in quinoid rings, C=C in benzenoid rings), 1303 cm^−1^ (C–N stretching), 1143 cm^−1^ (C=N stretching vibrations), 822 cm^−1^ (out-of-plane deformation of C–H in the 1, 4-disubstituted benzene ring) and 510 cm^−1^ (aromatic ring deformation of C–H). In the FTIR spectra of the AS-g-PAni@MS nanocomposite, for MSNs, the characteristic peaks are 3357(–OH) and 1120 (Si–O–Si), ascorbic acid 3207 (–OH), 1743 (C=O), 1693 (C–O stretching), 1276 (C–O–C stretching), 753 and 579 (–OH out of plane deformation) [25,52], while PAni peaks are observed at 1551, 1468 (quinoid and benzenoid C=C stretching), 1349 (C–N stretching) and 845 (C–H Benzene deformation peaks) with deviations. One new peak at 2367 cm^–1^ is due to formation of oxime bond (–NH–O–) between ascorbic acid and PAni. The interaction between MSN and blend of AS-PAni was analyzed by the formation of hydrogen bonding between silanol groups on silica and π–electron cloud of PAni which resulted in shift of quinoid and benzenoid stretching frequency to 1551 and 1468 cm^–1^ from 1574 and 1496 cm^–1^ [22,53,54,55]. The FTIR spectra of the AS-g-PAni@MS nanocomposite after adsorption (Supplementary Materials Figure S1 of CV dye also supports the involvement of surface active –OH/–NH_2_ groups in removal mechanism.

**Figure 3:**
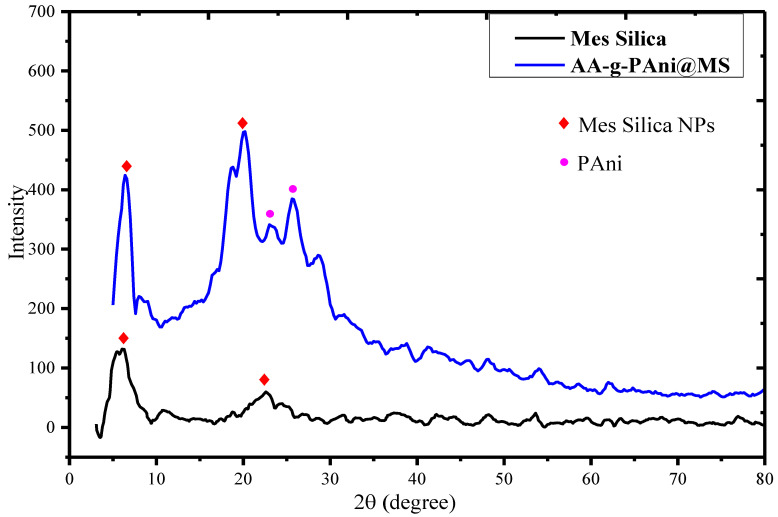
XRD spectra of mesoporous silica nanoparticles (black line) and AS-g-PAni@MS nanocomposite (blue line).

Figure 3 represents the XRD spectra of MSNs (black line) and AS-g-PAni@MS (blue line) in the 2θ range of 5–80°. As can be seen from Figure 3 (black line), the XRD spectra of MSNs show the characteristics peaks at 2θ value of 5.80° and 22.12° [14], while the XRD of the spectra represents the characteristics peaks of both MSNs and PAni at 6.31° (MSNs) and 20.09°, 25.69° for periodicity parallel and perpendicular to PANI chains. Moreover, a broad peak at 2θ = 25.69 indicates the characteristic distance between the ring planes of the benzene ring to the adjacent chains [56]. The XRD spectra indicate the more or less amorphous nature in the material due to the presence of AS-g-PAni polymer blend, which reduces the intensity of the peaks. More information about the crystallite size can be obtained by using the Scherrer equation, as given by Equation (19) [57]:

(19) $$D=\frac{0.9\lambda }{\beta \cos\theta }$$

where *D* is the crystal’s size, *λ* is the wavelength used (i.e., 1.54 A°), *β* is the half-width of the most intense peak and *θ* is the angle of diffraction.

Equation (19) reveals that the average crystallite size of both MSNs and AS-g-PAni@MS was 35.71 and 25.43 nm, respectively. As noted in Figure 3, there is a great compression in the peak width and intensity due to the functionalization of MSNs with AS-g-PAni polymer chains. These interactions are vital for facilitating variations of the d-spacing and lattice distortions during aggregation, resulting in additional size compressions from 35.71 nm to 25.43 nm after functionalization [58]. Similar trend for particle size for MSNs and functionalized MSNs was also reported by Lin et al., Das et al. and Dogra et al. [59,60,61]. The XRD data show that the surface of MSNs was successfully modified by the AS-g-PAni polymer chains, resulting in well-dispersed, semi-crystalline solids with adequate functional density.

The thermal stability of the synthesized material was assessed by the application of thermogravimetric analysis (TGA) followed by differential scanning calorimetry (DSC). Supplementary Materials Figure S2 represents the TGA–DSC curve of AS-g-PAni@MS nanocomposite which exhibited a two-stage weight loss for the material. The initial weight loss of 0.55% around 94 °C is suggest the loss of bound water molecules to the surface of material. The second stage in weight loss about 91.67% happens to be in temperature range of 98 to 503 °C. In this stage the degradation of carbonic constituents from benzene and ascorbic acid rings of the material including the breakage of Si–OH bonds and formation of Si–O–Si bonds takes place [62]. The behavior of the TGA curve is also supported by DSC curve as endothermic peak around 419 °C for thermal degradation of benzenoid carbon chains of PAni and ascorbic acid while the exothermic peak at 448 °C for formation of Si–O–Si bonds. Thus, reinforcement of 0.12% of MSNs into AS-g-PAni copolymer matrix provided good thermal stability for extreme temperature conditions.

**Figure 4:**
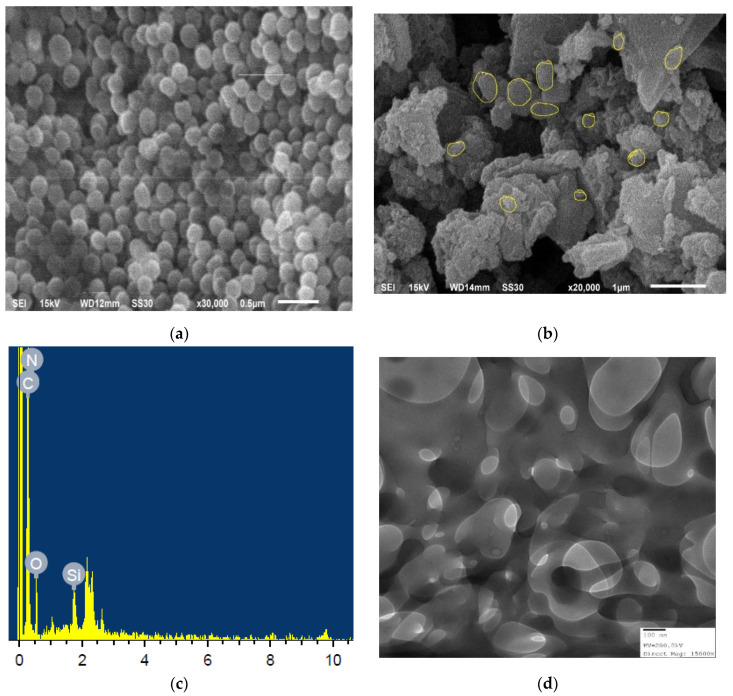

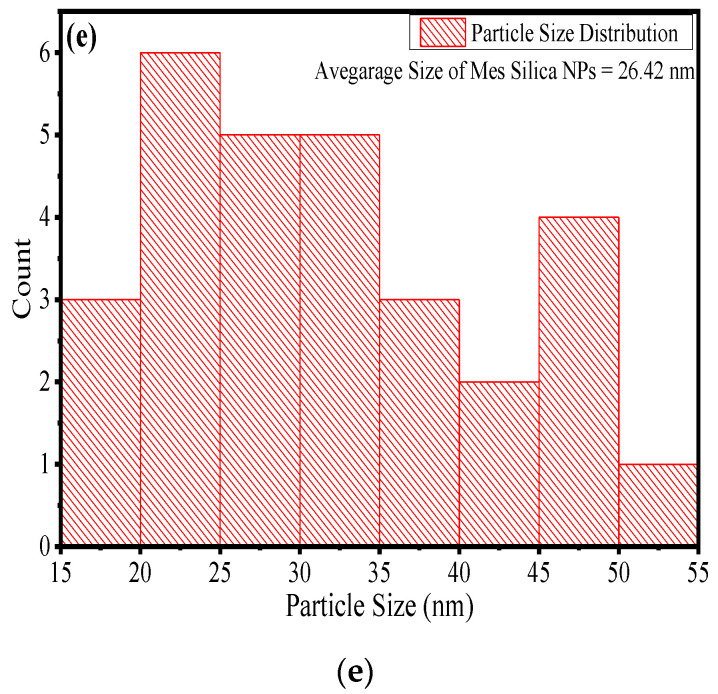
SEM image of (**a**) bare MSNs and (**b**) AS-g-PAni@MS nanocomposite. (**c**) EDX spectra showing individual constituent elements comprising the material. (**d**) TEM image of AS-g-PAni@MS nanocomposite showing the distribution of MSNs in the polymer matrix at 100 nm magnification range. (**e**) Gaussian distribution of particle size for assessing the average particle size of nanoparticles.

Scanning Electron Microscopy (SEM) was employed to observe the of surface morphological changes in the material during the solid-state reactions. Figure 4a constitutes the SEM image of bare MSNs which reflect a spherical type morphology. Figure 4b represents the SEM image of AS-g-PAni@MS nanocomposite which exhibits a flaky type of structure owing to functionalization with blend of AS-g-PAni with loosely agglomerated distribution of MSN particles on the surface (white dots). Further, the weight percentage of individual constituents used for the formation of AS-g-PAni@MS was observed by the energy dispersive X-rays (EDX) and is given in the Figure 4c. The total output and conclusion received by EDX analysis expresses the composition of AS-g-PAni@MS as C (52.53%), N (20.30%), O (25.69%) and Si (1.49%). Figure 4d represents the TEM image of synthesized AS-g-PAni@MS nanocomposite in which spherical elongated small sized particles are observed with an ordered distribution along the AS-g-PAni polymer matrix. The TEM image also supports the porous matrix formation of the material. Figure 4e was utilized to obtain the average particle size of MSNs in the polymer matrix of AS-g-PAni, using statistical domain tools like Gaussian distribution. With a frequency of 6% the average particle size was estimated as 26.42 nm, which is in close concurrence with XRD results (25.43 nm). A similar trend for particle size for MSNs and functionalized MSNs was also reported by Lin et al., Das et al. and Dogra et al. [59,60,61].

**Figure 5:**
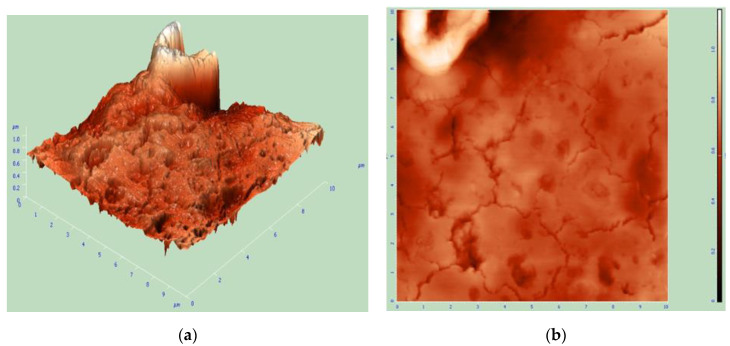
(**a**) 3D topographical atomic force microscopic (AFM) image and (**b**) 2D AFM topographical image of AS-g-PAni@MS nanocomposite.

To further explore the surface morphological variation of material after the in situ polymerization reaction, a more advanced analytical technique called as atomic force morphology (AFM) was utilized. The primary purpose of this morphology to assess the surface roughness with respect to nanoparticle reinforcement verified by statistical data. The 3D and 2D AFM morphological images of the synthesized AS-g-PAni@MS nanocomposite are given in Figure 5a,b. The obtained value of surface roughness of 75.71 nm after analysis suggested the efficient functionalization and stabilization of MSNs with copolymer chain of AS-g-PAni. In Figure 5b, the occurrence of small well-distributed dots indicates a well-ordered distribution of MSN in AS-g-PAni matrix with a root mean square value of 115.70 nm, and cracks are supporting the porous structure of the matrix with surface skewness value of −0.12.

Zeta sizer (Malvern) was utilized for the determination of point of zero charge (pHpzc) to explain the effect of pH of the medium on the adsorption of CV by the synthesized nanocomposite material. Particle suspension was made by pouring out 20 mg material in 20 mL of 0.1 M KCl solution under sonication with variable pH from 1 to 10. Zeta potential values were determined at the interval of 1 pH units in the pH range from 1 to 10, and obtained results are depicted in Supplementary Materials Figure S3. Zeta potential values were decreased from 35.30 mV at pH of 1 to −39.78 mV at a pH of 10 with zero potential at pH of 4.45. This point of zero potential where the charge on the surface is zero is called an isoelectric point (IEP) or point of zero charge, and the corresponding pH is termed as pH_iep_ or pH_pzc_.

### 3.2. BBD Design and Analysis of Variance (ANOVA)

The experimental design was constructed by using Design Expert 10.0 software (State-ease, Minneapolis, MN, USA), to optimize the four aforementioned operational parameters known to affect the adsorption of CV on AS-g-PAni@MS nanocomposite. As noted in Supplementary Materials Table S1, these three variables, namely the sonication time (A) (60–120 min), CV solution pH (B) (4–7), CV concentration (C) (30–60 mg L^−1^) and adsorbent dose (D) (20–40 mg), were selected based primarily on batch-based experiments. Quadratic regression modeling is employed to determine the responses of the respective coded values for the four variables, which, in turn, are based on the experimental and theoretical outcomes given by Equation (20):

R (mg g^−1^) = +18.95 − 0.72 × A + 3.74 × B + 5.35 × C − 5.87 × D − 0.98 × AB + 0.46 × AC + 3.12 × AD − 0.64 × BC − 3.84 × BD − 0.71 × CD − 0.77 × A^2^ − 0.74 × B^2^ − 0.42 × C^2^ + 2.40 × D^2^ (20)

The statistical implication and interaction results of each term obtained from the quadratic model are manifested via the analysis of variance (ANOVA), as shown in Table 1. The respective coefficient terms and the significance of the regression model are evaluated by the P and F values, using Fisher’s null hypothesis method. Here, increased applicability is associated with the quadratic relevance model, and each coefficient term is imposed by the small *p* and large F value. The large F and small P values confirm the model’s appropriateness, as evidenced by the RSM-coupled BBD [24]. The condition proposed by Fisher *p* > F < 0.05 can be seen in Table 1. Here, the reasonable *p* > F value of 0.0025 noted in the proposed quadratic regression model is statistically significant and relevant for the adsorption of CV on AS-g-PAni@MS nanocomposite. Linear variable terms like the sonication time (A, *p* > F = 0.5411) are not significant while the CV solution’s pH (B, *p* > F = 0.0056), CV concentration (C, *p* > F = 0.0004) and adsorbent dose (D, *p* > F = 0.0002) are statistically significant. Based on the ANOVA analysis, the quadratic Equation (20) was further optimized by omitting the non-significant variables for which value of *p* > F is higher than 0.05. When only the statistically significant terms in Equation (20) are taken into consideration, we obtain Equation (21):

R1 = +18.95 +3.74 × B +5.35 × C − 5.87 × D − 0.98 × AB − 3.84 × BD − 0.74 × B^2^ (21)

Supplementary Materials Figure S4a represents the normal probability plot for acquiring the approximation of real system by regression model. As can be seen in Supplementary Materials Figure S2a, the points dispersed across the straight line without response portrays an appropriation curve of residuals. A scheme was imposed between the predicted values and actual values obtained by the experimental designs and can be portrayed by Supplementary Materials Figure S4b. The standard deviation (SD) of model was found to be 3.25 with a correlation values of R^2^ and R^2^_adj_ as 0.94 and 0.86, individually indicating that there is a correlation between the theoretical and experimental values of the adsorbent reaction data.

### 3.3. Interpretation of the 3D Surface and Optimization Plots

**Figure 6:**
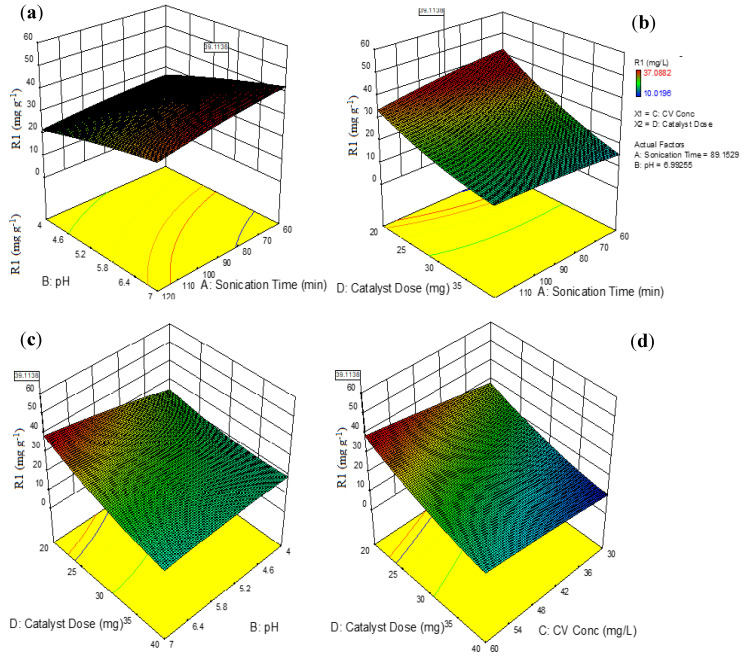

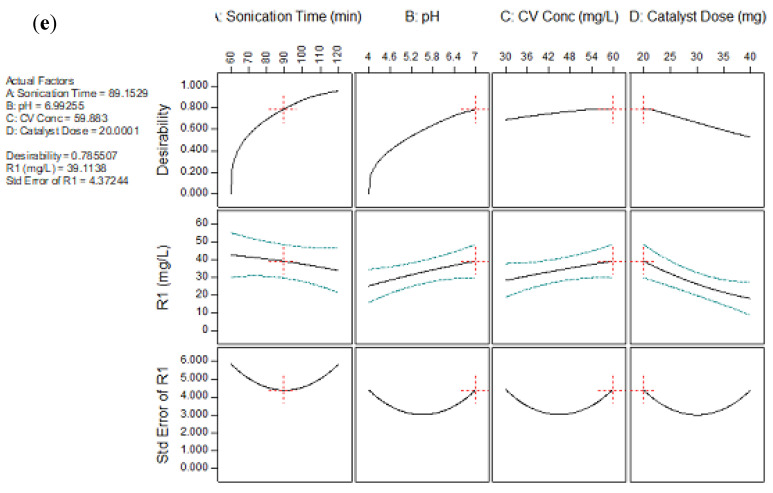
Three-dimensional surface interactive curves for (**a**) sonication time×pH of solution, (**b**) sonication time × adsorbent dose, (**c**) pH of solution × adsorbent dose and (**d**) CV concentration × catalyst dose. (**e**) Optimization plot for individual variable effecting the adsorption reaction.

The 3D surface designs are the graphic representations of the quadratic regression equation that describe the synchronous effect of two variables on the photodegradation reaction when the other variables are maintained. Figure 6a depicts the 3D surface interaction curve between the pH of the CV solution and the sonication time keeping other variables constant. Notably a gradual increase in the reaction time from 60 to 90 min accompanied with pH value from 4 to 7 results in gradual increase in adsorption capacity form 10.02 mg g^−1^ to 37.09 mg g^−1^. It can be inferred from Figure 6a that long radiation times and high pH values (>6) favor the adsorption of CV by AS-g-PAni@MS nanocomposite. The reason behind this behavior may be attributed to the large number of active pore sites on the surface that facilitate extensive host–guest interactions. As the reaction proceeds, increasingly more active sites become engaged in the adsorption of CV, resulting in a higher adsorption capacity under longer sonication times [35]. There seems not much increase in adsorption capacity from 90 to 120 min which suggested 90 min as optimized time for adsorption reaction. Supplementary Materials Figure S3 depicts the zeta potential curve which suggested the point the charge value (pH_pzc_) as 4.45 which means that at pH > pH_pzc_ (4.45) the surface of the adsorbent will be negative and pH < pH_pzc_ (4.45) the surface will be positive. At pH 4, the positive surface of adsorbent will exert a repulsive force the cationic CV molecule which resulted in low adsorption capacity. With a further increase in pH value to 5.5 and 7, the surface of the adsorbent will turn to a more negative magnitude which exerts high electrostatic forces to bind with cationic CV molecules, and as a result, adsorption capacity is increased [23].

Figure 6b shows the 3D surface interactive curve for sonication time and adsorbent dose. It can be inferred from figure that high sonication time and low adsorbent dose favored high adsorption capacity. With the increase in adsorbent dose from 20 to 40 mg, there seemed to be a gradual decrease in adsorption capacity from 37.09 to 10.02 mg g^−1^. Thus, as with the increase in nanocomposite adsorbent dose, hindrance occurs to the agglomeration of particles at a higher dose, which blocks the susceptibility of CV dye molecules to the bulk of the material layer, and as result, adsorption capacity decreases [23]. Figure 6c represents the 3D surface interactive curve for adsorbent dose and pH of the solution. The information extracted from Figure 6c supported the favorability of high pH and low adsorbent dose for high adsorption capacity. The two variable interactions (adsorbent dose×CV concentration) on the adsorption capacity of synthesized material are given by Figure 6d. As can be inferred from the figure, high CV concentration (60 mg L^−1^) and low adsorbent dose were found to be favorable for the high efficiency of the adsorption process. With the increase in CV concentration from 30 to 60 mg L^−1^ with respect to adsorbent dose (20 to 40 mg), there is a gradual increase in adsorption capacity occurred [29]. As the concentration increases, more and more substrate molecules are available to penetrate through the solid/liquid interface, and as a result, higher efficiency is observed. Figure 6e represents the optimization ploy with desirability for individual variable accompanied by standard error value. In Figure 6e, the optimized values of individual parameters assessed by BBD design are given as sonication time (89.15 min), solution pH (6.99), CV concentration (59.88 mg L^−1^) and adsorbent dose (20.00 mg), with desirability of 0.79 and an SD of 4.37 in 95% confidence interval.

### 3.4. Adsorption Isotherms and Thermodynamics

**Figure 7:**
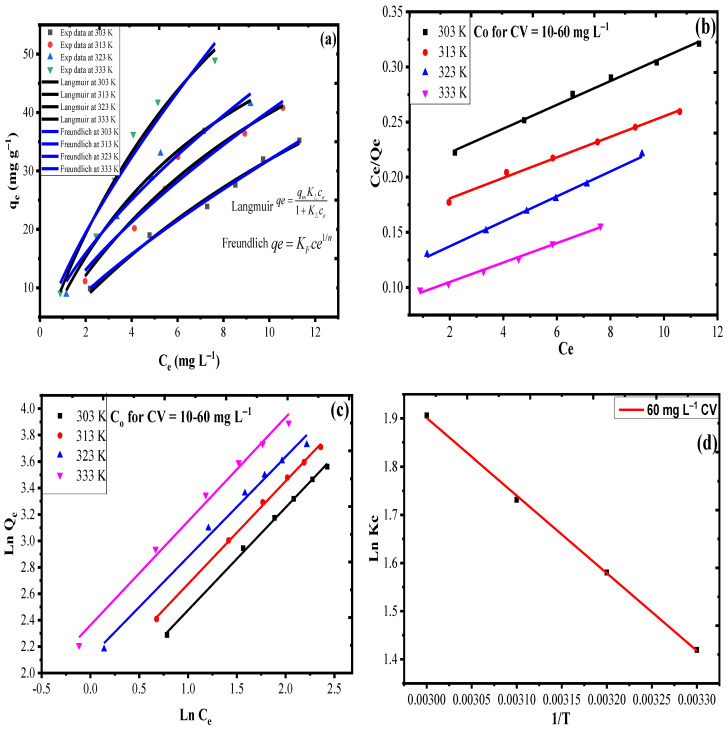
(**a**) Ce vs. q_e_ plot for adsorption isotherm data. Linear regression plots for (**b**) Langmuir, (**c**) Freundlich and (**d**) Ln Kc vs. 1/T thermodynamic plot at 303–333 K.

Adsorption isotherm studies were performed, using a 20 mL aliquot of 10, 20, 30, 40, 50 and 60 mg L^−1^ CV concentration at pH 6.9 for an optimum sonication time of 90 min, using 20 mg AS-g-PAni@MS nanocomposite in a temperature range of 303–333 K. The data obtained after experiment were applied to the Langmuir model (Equation (6)) and the Freundlich model (Equation (7)). Figure 7b,c represents the Langmuir and Freundlich plot obtained through linear regression method. Table 2 consist of all the valuable information extracted from slope and intercept of these plots. The suitability of any model to explain the experimental data with minimum error depends upon the higher value of regression coefficient (R^2^) and lower value of root mean square error value (RMSE). It can be inferred from Table 2 that the Langmuir model with high R^2^ and low RMSE values (0.99, 0.003) at 303 K, (0.99, 0.003) at 313 K, (0.99, 0.002) at 323 K and (0.99, 0.002) at 333 K is found to be found to be more suitable to explain the adsorption process as compared to the Freundlich model (0.98, 0.02) at 303 K, (0.98, 0.02) at 313 K, (0.98, 0.06) at 323 K and (0.98, 0.06) at 333 K. The suitability of the Langmuir model suggested the monolayer formation by CV molecules on the surface of AS-g-PAni@MS and the maximum monolayer adsorption capacity (q_m_) of the synthesized material was found to be 88.42 mg g^−1^ at 303K, 92.51 mg g^−1^ at 313 K, 107.41 mg g^−1^ at 313 K and 113.25 mg g^−1^ at 333 K. The value of Langmuir constant also called as affinity constant (K_L_) 0.054, 0.057, 0.099, and 0.101 L mg^−1^ at 303–333 K also supports the high affinity of CV molecules towards the adsorbent surface at a higher temperature. The value of n calculated by the Freundlich model 1.27, 1.28, 1.29, 1.32 at 303–313 K is greater than 1, suggesting that the CV adsorption on the material surface is favorable. The verification of the data obtained through linear regression was done through a comparison with data obtained through nonlinear natural adsorption isotherm curves for both Langmuir and Freundlich given in Figure 7a. The data obtained through non-linear regression given in Supplementary Materials Table S3 were found to be in great concurrence with the data assessed by linear regression.

Figure 7d and Table 3 constitute the thermodynamic plot and corresponding calculated parameters obtained through adsorption experiment conducted for 60 mg L^−1^ CV in temperature range of 303–333 K. Based on Equations (8)–(10), a value of enthalpy (ΔH) as 3.62 KJ mol^−1^ with positive magnitude suggested the endothermic nature of the adsorption reaction of CV with AS-g-PAni@MS which deals with an entropy of 0.015 KJ mol^−1^ K^−1^. The feasibility and spontaneous nature of the adsorption reaction was analyzed by Gibbs free energy (ΔG) values. The values of ΔG −0.99 KJ mol^−1^ at 303 K, −1.08 KJ mol^−1^ at 303 K, −1.23 KJ mol^−1^ at 303 K and −1.38 KJ mol^−1^ at 303 K suggested the adsorption reaction is spontaneous and feasible and the increase in negative magnitude of ΔG values with increase in temperature suggested the high feasibility of the adsorption reaction of CV with AS-g-PAni@MS nanocomposite.

### 3.5. Adsorption Kinetics

**Figure 8:**
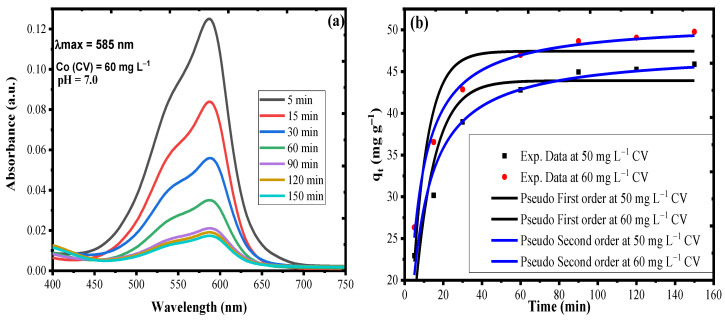
(**a**) Time-dependent UV–Vis spectra for CV adsorption. (**b**) Non-linear kinetic plot for pseudo first order (black line) and pseudo second order (blue line).

Figure 8a represents the time-dependent UV–Vis spectra for CV adsorption on nanocomposite material. It can be inferred from the graph, that with passage of time, the magnitude of absorption by CV molecule continuously decreased from 0.125 at 5 min to 0.021 at 90 min, due to adherence of CV molecule on the surface of the nanocomposite material. After 90 min, the very appreciable change in absorbance value suggested that the surface of the adsorbent was saturated with the CV molecules and no surface-active site was available. The kinetic studies were performed by using a 20 mL aliquot of 50 and 60 mg L^−1^ CV at pH 6.9, for sonication time of 5–150 min, using 20 mg AS-g-PAni@MS nanocomposite at 333 K. The data obtained after experiment were applied to pseudo first order (Equation (11)) and pseudo second order (Equation (12)). Figure 8b represents the pseudo first and second order plots obtained through non-linear regression method. Table 4 consist of all the key kinetic variables required to define the mechanism of the adsorption reaction obtained through nonlinear regression. It can be inferred from the Table 4, pseudo second order with high R^2^ and low RMSE values (0.96, 2.93) at 50, and (0.99, 0.86) at 60 mg L^−1^ CV concentration is found to be more suitable to explain the adsorption mechanism, as compared to pseudo first order (0.84, 3.55) at 50 and (0.86, 3.25) at 60 mg L^−1^ CV concentration. The suitability of pseudo second order model suggested that the CV molecules are interacting with adsorbent surface through pure covalent bonds, i.e., coordination bond between NH/OH groups of the adsorbent with CV^+^ ions. Moreover the q_e,cal_ and q_e,exp_ values for the pseudo second order had a higher correlation (Δq = 1.64 for 50 and 1.25 for 60 mg L^−1^ CV) as compared to the pseudo first order (Δq = 1.95 for 50, 2.32 for 60 mg L^−1^ CV). The rate constants for pseudo first and second order models were found to be K_1_= 0.099 min^–1^ for 50 and 0.126 min^–1^ for 60 mg L^–1^ CV, and K_2_ = 0.003 L mg^–1^ min^–1^ for 50 and 0.003 L mg^–1^ min^–1^ for 60 mg L^–1^ CV.

### 3.6. Breakthrough Studies in Fixed Bed Method and Application of the Thomas Model

**Figure 9:**
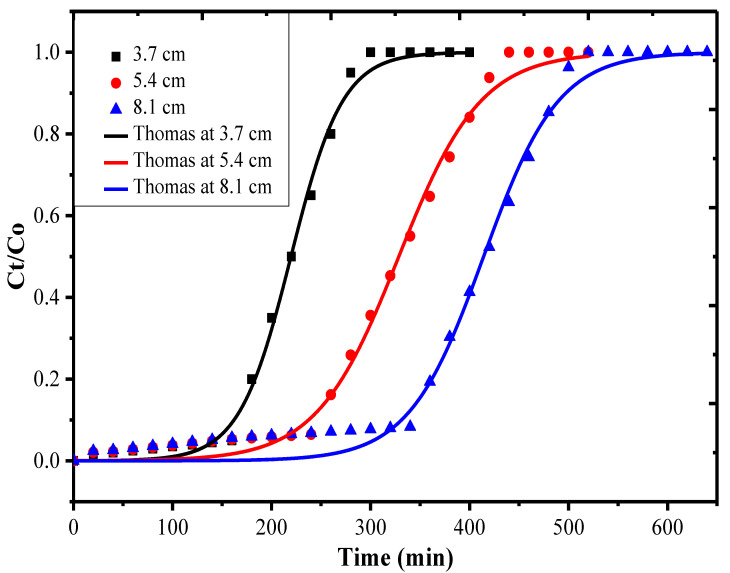
Break curves associated with Thomas model nonlinear fitting at 3.7 cm (black line), 5.4 cm (red line) and 8.1 cm (blue line) bed heights for CV adsorption on AS-g-PAni@MS nanocomposite.

Based on the fixed bed experiment design for breakthrough studies outlined in Section 2.8, the break curve is given in Figure 9 for the bed heights 3.7, 5.4 and 8.1 cm. The various operation parameters calculated after using Equations (13)–(16) are given in Table 5. It can be inferred from Table 5 that the estimated *q_eq_* was found to be 91.67 mg g^−1^ at 3.7 cm, 87.24 mg g^−1^ at 5.4 cm and 81.58 mg g^−1^ for 8.1 cm bed height. It was observed that, with increase in bed height, the adsorption capacity or % removal decreased from 91.67% at 3.7 cm to 81.58% at 8.1 cm. The decrease in % removal value is attributed towards the higher amount of the AS-g-PAni@MS nanocomposite (*m_ads_*) at higher bed heights, which posed a higher diffusion path length for the CV molecules to pass through [47]. The increase in diffusion path length leads to an increase in *t_b_*, *t_e_, V_eff_* and Z_m_ values. The passage of CV molecules from liquid to solid surface, and then along the solid bed layer and again to outlet, requires a larger time [49]. Thus, the values various other operation parameters were found to be as breakthrough time (*t_b_*) as 93.9, 123.1, and 209.4 min; total exhaustion time (*t_e_*) 360.2, 517.2, and 620.5 min; mass transfer coefficient (Z_m_) 2.74, 4.11, and 5.37 cm; and Z_m_/L 0.74, 0.76, 0.66 for 3.7, 5.4 and 8.1 cm bed heights.

The verification of the breakthrough data was achieved by the application of a dynamic Thomas model. The values estimated by Equation (17) are given in Table 6. It is observed that the data were well defined by the Thomas model, based on high value of regression coefficient (R^2^) and low value of RMSE given as (0.99, 0.02) for 3.7 cm, (0.99, 0.03) for 5.4 cm and (0.99, 0.04) for 8.1 cm bed height. The estimated stoichiometric adsorption capacity (q_eq_) values 87.79, 84.37, and 79.48 mg g^−1^ were also found to be in close concurrence with the fixed bed breakthrough data. The dynamic Thomas rate constant K_TH_ (mL mg^−1^ min^−1^) was found to be 0.64, 0.41, and 0.28, suggesting that K_TH_ is inversely proportional to the bed height, and also supporting the % CV removal data obtained by fixed bed studies.

### 3.7. Regeneration and Reusability of the Adsorbent

**Figure 10:**
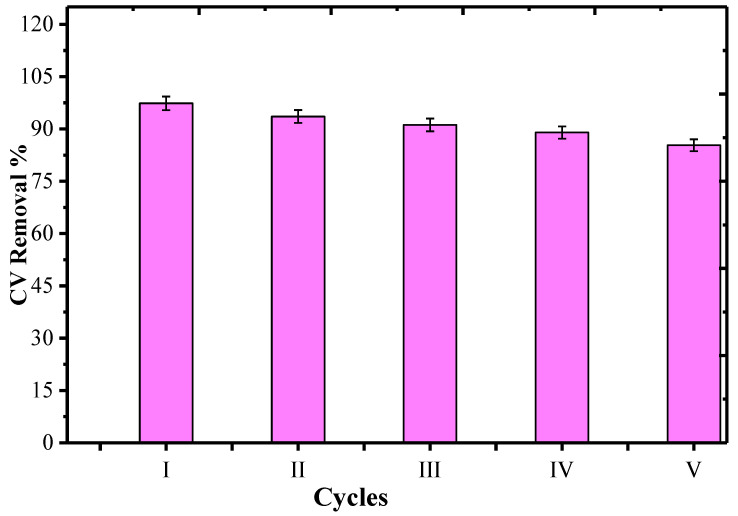
The regeneration cycles of AS-g-PAni@MS bonded CV, using 0.1 M HNO_3_ solution.

One of the important problems associated with adsorption technology is the safe disposal of the spent adsorbent. This hurdle was later on overcome by researchers through the regeneration process, which involves the recovery of the spent adsorbent for reuse that, in a way, reduces the cost of the experiment [63]. In the present study, the regeneration process was done by solvent washing method in which the 20 mg CV–AS-g-PAni@MS was mixed with 20 mL of 0.1 M HNO_3_ solution and placed under sonication equipped with a magnetic stirrer until all the CV molecules became detached from the AS-g-PAni@MS. The material was collected by filtration and washed by distilled water until the effluent pH became neutral and dried in a hot-air oven for 2 h at 60 °C. The recovered adsorbent was again utilized for the adsorption process of CV at the optimized conditions, and results are given in Figure 10. In the first cycle, the CV removal % was 97.11%; repeating the same procedure of eluting of the CV molecules from adsorbent surface, the CV removal % for second, third, fourth and fifth cycle was found to be 93.21%, 91.40%, 88.80% and 85.17%. In five cycles, only a decrement of 13% in adsorbent capacity suggested that the material is highly efficient for treatment of water contaminated by CV.

### 3.8. Comparison with Literature

A comparison of adsorption efficiency of the synthesized material with other reported material for CV removal was attained. The data given in Table 7 suggested that the present study was highly efficient with a 97.11% of CV removal as compared to other reported materials in the same domain.

### 3.9. Comparison with Individual Constituents

A comparison of the removal efficiency (%) of synthesized material with its individual constituents for the adsorption of CV dye was examined using the optimized reaction conditions. Supplementary Materials Figure S5a represents the UV–Vis spectra for CV pertaining to AS, MSNs, PAni, AS-g-PAni and AS-g-PAni@MS. It can be inferred that the absorbance values for CV after adsorption was found to be 1.24 for AS, 0.84 for MSNs, 0.55 for PAni, 0.11 for AS-g-PAni and 0.02 for AS-g-PAni@MS, respectively. The corresponding CV removal rate was found to be 42.75%, 61.23%, 74.62%, 94.92% and 97.45%, suggesting that the synthesized material is most efficient as compared to its individual constituents. The enhanced efficiency in the AS-g-PAni matrix after reinforcement of MSNs at pH 7 may be due to interactions between silanol groups and π-electron cloud of PAni phenyl group that resulted in an ordered distribution of MSNs and exerted enhanced thermal, chemical and adsorption properties.

## 4. Conclusions

In the present study, mesoporous silica nanoparticles (MSNs) synthesized through a sol–gel process and calcined at 600 °C were further surface functionalized by a copolymer chain of L-ascorbic acid (AS) and polyaniline (PAni) by in situ free radical oxidative polymerization reaction. The surface modification of MSNs by AS-g-PAni was confirmed by using various analytical techniques, like FTIR, XRD, SEM-EDX, TEM and AFM. The composition of AS-g-PAni@MS was found to be composed of C (52.53%), N (20.30%), O (25.69%) and Si (1.49%), with 26.42 nm as particle size. Further, it was applied for the adsorption of crystal violet (CV) dye under batch, as well as fixed bed method. RSM–BBD was taken into consideration, to optimize the various operational parameters effecting the adsorption through batch method. To explore maximum efficiency of the material, it was further subjected to adsorption of CV under fixed bed method, using variable bed heights such as 3.7, 5.4 and 8.1 cm. Based on high value of regression coefficient (R^2^) and low value of RMSE given as (0.99, 0.02) for 3.7 cm, (0.99, 0.03), the breakthrough data were very well defined by the Thomas model with optimum concurrence of stoichiometric adsorption capacity values. The external mass transfer equilibrium data were well fitted by the Langmuir model with maximum monolayer adsorption capacity of 88.42 mg g^−1^ at 303K, 92.51 mg g^−1^ at 313 K, 107.41 mg g^−1^ at 313 K and 113.25 mg g^−1^ at 333 K. The uptake of CV by AS-g-PAni@MS was well defined by pseudo second order model, with rate constant K_2_ = 0.003 L mg^–1^ min^–1^ for 50 and 0.003 L mg^–1^ min^–1^ for 60 mg L^–1^ CV. The adsorption reaction was endothermic, with an enthalpy (ΔH) value of 3.62 KJ mol^-1^, and highly efficient for treatment of CV-contaminated water for more the five consecutive cycles. The future implication of this study is to dope the mesoporous silica with metal nanoparticles like Ag, Au, Fe, Zn, etc., and to explore their impact on the photodegradation of low-density polyethylene for H_2_ generation for renewable energy purposes.

## Figures and Tables

**Table 1:** Analysis of variance (ANOVA) for the adsorption of crystal violet (CV) on AS-g-PAni@MS.

Source	Sum of Squares	df	Mean Square	F Value	*p*-Value Prob > F	
Model	1093.30	14	78.09	4.97	0.0025	significant
A-Sonication Time	6.16	1	6.16	0.39	0.5411	
B-pH	167.76	1	167.76	10.68	0.0056	
C-CV Conc	343.69	1	343.69	21.89	0.0004	
D-Catalyst Dose	412.81	1	412.81	26.29	0.0002	
AB	3.81	1	3.81	0.24	0.0063	
AC	0.83	1	0.83	0.053	0.8213	
AD	39.06	1	39.06	2.49	0.1370	
BC	1.65	1	1.65	0.11	0.7506	
BD	59.04	1	59.04	3.76	0.0072	
CD	2.01	1	2.01	0.13	0.7256	
A^2^	3.87	1	3.87	0.25	0.6273	
B^2^	3.56	1	3.56	0.23	0.0064	
C^2^	1.12	1	1.12	0.072	0.7929	
D^2^	37.41	1	37.41	2.38	0.1450	
Residual	219.81	14	15.70			
Lack of Fit	196.94	10	19.69	3.44	0.1223	not significant
Pure Error	22.87	4	5.72			
Cor Total	1313.11	28				

AB (Sonication Time × pH), AC (Sonication Time × CV Conc.), AD (Sonication Time × Catalyst Dose), BC (pH × CV Conc.), BD (pH×Catalyst Dose), CD (CV Conc. × Catalyst Dose).

**Table 2:** Adsorption isotherm parameters obtained for the adsorption reaction of CV with AS-g-PAni@MS nanocomposite at 303–333 K.

Isotherm Model	Equations	Parameters	303 K	313 K	323 K	333 K
Langmuir	$\frac{C_{e}}{q_{e}}=\frac{1}{{}_{\;}q_{m}K_{L}\;\;}+\frac{C_{e}\;}{q_{m}}$	*q_m_* (mg g^-1^)	88.42	92.51	107.41	113.25
		*K_L_* (L mg^-1^)	0.054	0.057	0.099	0.101
		R^2^	0.99	0.99	0.99	0.99
		RMSE	0.003	0.003	0.002	0.002
Freundlich	$\ln q_{e}=\frac{1}{{}_{\;}n\;\;}\ln C_{e}+\ln K_{F}$	*K_F_* (mg g^−1^) (L mg^−1^)^1/*n*^	5.46	6.65	8.34	10.62
		n	1.27	1.28	1.29	1.32
		R^2^	0.98	0.98	0.98	0.98
		RMSE	0.02	0.02	0.06	0.06

*q_m_* (maximum adsorption capacity), *K_L_* (Langmuir constant), R^2^ (regression coefficient), RMSE (root mean square error), *K_F_* (Freundlich adsorption capacity), n (Freundlich constant for favorability).

**Table 3:** Thermodynamic parameters for adsorption reaction of CV with AS-g-PAni@MS nanocomposite.

Model	Enthalpy (ΔH) KJ mol−1	Entropy (ΔS) (KJ mol−1 K−1)	Gibbs Free Energy (ΔG) (KJ mol−1)			
			303 K	313 K	323 K	333 K
Gibbs	3.62	0.015	−0.99	−1.08	−1.23	−1.38

**Table 4:** Kinetics parameters obtained for the adsorption reaction of CV (50 and 60 mg L^−1^), with AS-g-PAni@MS nanocomposite at 333 K.

Kinetic Model	Equation	Parameters	50 mg L^−1^	60 mg L^−1^
Pseudo First	$q_{t}=q_{e}(1-e^{-k_{1}t})$	q_e,exp_	45.87	49.76
		q_e,cal_	43.92	47.44
		*K*_1_ (min^−1^)	0.099	0.126
		R^2^	0.84	0.86
		RMSE	3.55	3.25
Pseudo Second	$q_{t}=\frac{k_{2}q_{e}{}^{2}t}{1+k_{2}q_{e}t}$	q_e,cal_	47.51	51.01
		*K*_2_ (L mg^−1^ min^−1^)	0.003	0.003
		R^2^	0.96	0.99
		RMSE	2.93	0.86

**Table 5:** Estimated operational variables from fixed bed studies for CV adsorption on AS-g-PAni@MS nanocomposite.

Model	Bed Height (cm)	q_eq_ (mg g^−1^)	%Removal	V_eff_ (L)	t_b_ (min)	t_e_ (min)	Z_m_ (cm)	Z_m_/L
Fixed Bed	3.7	99.06	91.67	3.6	93.9	360.2	2.74	0.74
	5.4	94.71	87.24	5.2	123.1	517.2	4.11	0.76
	8.1	86.8	81.58	6.2	209.4	620.5	5.37	0.66

**Table 6:** Estimated operational variables extracted by the Thomas model for CV adsorption on AS-g-PAni@MS nanocomposite.

Model	Parameters	Bed Height		
		3.7 cm	5.4 cm	8.1 cm
Thomas Model	q_eq_ (mg g^−1^)	87.79	84.37	79.48
	K_TH_ (mL mg^−1^ min^−1^)	0.64	0.41	0.28
	R^2^	0.99	0.99	0.99
	RMSE	0.02	0.03	0.04

q_eq_ (estimated stoichiometric adsorption capacity), K_TH_ (dynamic Thomson rate constant), R^2^ (regression coefficient), RMSE (root mean square error).

**Table 7:** Contains the comparison of the synthesized material with the materials reported in the literature for CV adsorption with respect to % removal, dye concentration and time.

Material	Target	Time (min)	Target Concentration (mg L^−1^)	% Removal	References
Dy_x_MnFe_2−x_O_4_ nanoparticles decorated over mesoporous silica	Crystal Violet	150	5	98	[14]
SiO_2_/NiFe_2_O_4_	Methyl Orange	60	10	5	[64]
MCM-41 mesoporous silica nanoparticles	Auramine O	20	20	56.43	[65]
MCM-41	Crystal Violet	120	600	90	[66]
MIL-68(Al)@SBA-15	Crystal Violet	30	50	85	[67]
CuO/MCM-41 nano composite	Crystal Violet	60	36	26.52	[68]
Ascorbic acid-g-Polyanile@Mes. Silica Nanocomposite	Crystal Violet	90	60	97.11	Present Study

## Supplementary Materials

The following are available online at https://www.mdpi.com/2079-4991/10/12/2402/s1. Table S1: Variable table containing the minimum, mean and maximum values of parameters affecting adsorption reaction. Table S2: Design of the experiments proposed by BBD model and their response. Figure S1: FTIR spectra of AS-g-PAni@MS nanocomposite after adsorption of CV dye. Figure S2: TGA-DSC curve to explain the thermal behaviour of AS-g-PAni@MS nanocomposite. Figure S3: Zeta potential curve to obtain the point of zero charge of the nanocomposite material. Figure S4: (a) Normal Probability Curve (b) Residual vs. Run plot obtained through RSM-BBD analysis. Table S3: Adsorption isotherm parameters obtained for the adsorption reaction of CV with AS-g-PAni@MS nanocomposite at 303–333 K obtained through nonlinear regression. Figure S5: (a) UV-Vis Spectra for CV adsorption by AS-g-PAni@MS and its individual constituents (b) removal rate (%) for AS, MSNs, PAni, AS-g-PAni and AS-g-PAni@MS nanocomposite for 60 mg L−1 CV at pH 7.

## Abbreviations

AS	L-Ascorbic Acid
PAni	Polyaniline
AS-g-PAni@MS	Ascorbic acid-grafted-Polyaniline mesoporous silica nanocomposite
FTIR	Fourier-transform Infrared
SEM	Scanning Electron Microscope
TEM	Transmission Electron Microscope
XRD	X-ray Diffraction
EDX	Energy-dispersive X-ray spectroscopy
DSC	Differential Scanning Calorimetry
TGA	Thermogravimetric analysis
AFM	Atomic Force Microscopy
UV–Vis	UV–Visible Spectroscopy
APS	Ammonium persulphate

## References

[1] Pongkitdachoti U., Unob F. (2018). Simultaneous Adsorption of Silver Nanoparticles and Silver Ions on Large Pore Mesoporous Silica. J. Environ. Chem. Eng..

[2] Cakić S.M., Valcic M.D., Ristić I.S., Radusin T., Cvetinov M.J., Budinski-Simendić J. (2019). Waterborne Polyurethane-Silica Nanocomposite Adhesives Based on Castor Oil-Recycled Polyols: Effects of (3-Aminopropyl)Triethoxysilane (APTES) Content on Properties. Int. J. Adhes. Adhes..

[3] Boonsomwong K., Genix A.C., Chauveau E., Fromental J.M., Dieudonné-George P., Sirisinha C., Oberdisse J. (2020). Rejuvenating the Structure and Rheological Properties of Silica Nanocomposites Based on Natural Rubber. Polymer.

[4] Özcan A., Hamid F., Özcan A.A. (2021). Synthesizing of a Nanocomposite Based on the Formation of Silver Nanoparticles on Fumed Silica to Develop an Electrochemical Sensor for Carbendazim Detection. Talanta.

[5] Régibeau N., Tilkin R.G., Compère P., Heinrichs B., Grandfils C. (2020). Preparation of PDLLA Based Nanocomposites with Modified Silica by in Situ Polymerization: Study of Molecular, Morphological, and Mechanical Properties. Mater. Today Commun..

[6] He Y., Shao L., Hu Y., Zhao F., Tan S., He D., Pan A. (2020). Redox and PH Dual-Responsive Biodegradable Mesoporous Silica Nanoparticle as a Potential Drug Carrier for Synergistic Cancer Therapy. Ceram. Int..

[7] Manzano J.S., Wang H., Kobayashi T., Naik P., Lai K.C., Evans J.W., Slowing I.I. (2020). Kinetics of the Functionalization of Mesoporous Silica Nanoparticles: Implications on Surface Group Distributions, Adsorption and Catalysis. Microporous Mesoporous Mater..

[8] Ren D., Xu J., Chen N., Ye Z., Li X., Chen Q., Ma S. (2020). Controlled Synthesis of Mesoporous Silica Nanoparticles with Tunable Architectures via Oil-Water Microemulsion Assembly Process. Colloids Surfaces A Physicochem. Eng. Asp..

[9] Du X., He J. (2011). Facile Fabrication of Hollow Mesoporous Silica Nanospheres for Superhydrophilic and Visible/Near-IR Antireflection Coatings. Chem. A Eur. J..

[10] Jaafar J.A., Kamarudin N.H.N., Setiabudi H.D., Timmiati S.N., Peng T.L. (2019). Mesoporous Silica Nanoparticles and Waste Derived-Siliceous Materials for Doxorubicin Adsorption and Release. Materials Today: Proceedings.

[11] Chen J., Sheng Y., Song Y., Chang M., Zhang X., Cui L., Meng D., Zhu H., Shi Z., Zou H. (2018). Multimorphology Mesoporous Silica Nanoparticles for Dye Adsorption and Multicolor Luminescence Applications. ACS Sustain. Chem. Eng..

[12] Sun M., Wang H., Li X. (2020). Modification of Cellulose Microfibers by Polyglutamic Acid and Mesoporous Silica Nanoparticles for Enterovirus 71 Adsorption. Mater. Lett..

[13] Niu B., Zhou Y., Wen T., Quan G., Singh V., Pan X., Wu C. (2018). Proper Functional Modification and Optimized Adsorption Conditions Improved the DNA Loading Capacity of Mesoporous Silica Nanoparticles. Colloids Surfaces A Physicochem. Eng. Asp..

[14] Baig M.M., Zulfiqar S., Yousuf M.A., Shakir I., Aboud M.F.A., Warsi M.F. (2021). Dy_x_MnFe_2−x_O_4_ Nanoparticles Decorated over Mesoporous Silica for Environmental Remediation Applications. J. Hazard. Mater..

[15] Li G., Lan J., Liu J., Jiang G. (2013). Synergistic Adsorption of As(V) from Aqueous Solution onto Mesoporous Silica Decorated Orderly with Al_2_O_3_ and Fe_2_O_3_ Nanoparticles. J. Colloid Interface Sci..

[16] Wang F., Huang W., Guo C., Liu C.Z. (2012). Functionalized Magnetic Mesoporous Silica Nanoparticles: Fabrication, Laccase Adsorption Performance and Direct Laccase Capture from Trametes Versicolor Fermentation Broth. Bioresour. Technol..

[17] Javdani H., Khosravi R., Etemad L., Moshiri M., Zarban A., Hanafi-Bojd M.Y. (2020). Tannic Acid-Templated Mesoporous Silica Nanoparticles as an Effective Treatment in Acute Ferrous Sulfate Poisoning. Microporous Mesoporous Mater..

[18] Gounani Z., Asadollahi M.A., Pedersen J.N., Lyngsø J., Pedersen J.S., Arpanaei A., Meyer R.L. (2019). Mesoporous Silica Nanoparticles Carrying Multiple Antibiotics Provide Enhanced Synergistic Effect and Improved Biocompatibility. Colloids Surfaces B Biointerfaces.

[19] Ebrahimpour M., Hassaninejad-Darzi S.K., Mousavi H.Z. (2020). Adsorption of Ternary Toxic Crystal Violet, Malachite Green and Methylene Blue onto Synthesised SBA-15 Mesoporous Nanoparticles. Int. J. Environ. Anal. Chem..

[20] Todorov A.R., Aikonen S., Muuronen M., Helaja J. (2019). Visible-Light-Photocatalyzed Reductions of N-Heterocyclic Nitroaryls to Anilines Utilizing Ascorbic Acid Reductant. Org. Lett..

[21] D’Amato C.A., Giovannetti R., Zannotti M., Rommozzi E., Minicucci M., Gunnella R., Di Cicco A. (2018). Band Gap Implications on Nano-TiO_2_ Surface Modification with Ascorbic Acid for Visible Light-Active Polypropylene Coated Photocatalyst. Nanomaterials.

[22] Mallakpour S., Soltanian S. (2016). Vitamin C Functionalized Multi-Walled Carbon Nanotubes and Its Reinforcement on Poly (Ester-Imide) Nanocomposites Containing L-Isoleucine Amino Acid Moiety. Compos. Interfaces.

[23] Hasan I., Walia S., Alharbi K.H., Khanjer M.A., Alsalme A., Khan R.A. (2020). Multi-Walled Carbon Nanotube Coupled β-Cyclodextrin/PANI Hybrid Photocatalyst for Advance Oxidative Degradation of Crystal Violet. J. Mol. Liq..

[24] Saad M., Tahir H., Khan J., Hameed U., Saud A. (2017). Synthesis of Polyaniline Nanoparticles and Their Application for the Removal of Crystal Violet Dye by Ultrasonicated Adsorption Process Based on Response Surface Methodology. Ultrason. Sonochem..

[25] Ahmad R., Hasan I., Mittal A. (2017). Adsorption of Cr (VI) and Cd (II) on Chitosan Grafted Polyaniline-OMMT Nanocomposite: Isotherms, Kinetics and Thermodynamics Studies. Desalin. Water Treat..

[26] Li Y., Yang C.X., Qian H.L., Zhao X., Yan X.P. (2019). Carboxyl-Functionalized Covalent Organic Frameworks for the Adsorption and Removal of Triphenylmethane Dyes. ACS Appl. Nano Mater..

[27] Li Y., Wang S., Shen Z., Li X., Zhou Q., Sun Y., Wang T., Liu Y., Gao Q. (2020). Gradient Adsorption of Methylene Blue and Crystal Violet onto Compound Microporous Silica from Aqueous Medium. ACS Omega.

[28] Gautam D., Hooda S. (2020). Magnetic Graphene Oxide/Chitin Nanocomposites for Efficient Adsorption of Methylene Blue and Crystal Violet from Aqueous Solutions. J. Chem. Eng. Data.

[29] Torabinejad A., Nasirizadeh N., Yazdanshenas M.E., Tayebi H.-A. (2017). Synthesis of Conductive Polymer-Coated Mesoporous MCM-41 for Textile Dye Removal from Aqueous Media. J. Nanostruct. Chem..

[30] Alotaibi N., Hammud H.H., Karnati R.K., Hussain S.G., Mazher J., Prakasam T. (2020). Cobalt-Carbon/Silica Nanocomposites Prepared by Pyrolysis of a Cobalt 2,2′-Bipyridine Terephthalate Complex for Remediation of Cationic Dyes. RSC Adv..

[31] Mani S., Bharagava R.N. (2016). Exposure to Crystal Violet, Its Toxic, Genotoxic and Carcinogenic Effects on Environment and Its Degradation and Detoxification for Environmental Safety. Reviews of Environmental Contamination and Toxicology.

[32] Wang H., Lai X., Zhao W., Chen Y., Yang X., Meng X., Li Y. (2019). Efficient Removal of Crystal Violet Dye Using EDTA/Graphene Oxide Functionalized Corncob: A Novel Low-Cost Adsorbent. RSC Adv..

[33] Chen C.Y., Kuo J.T., Yang H.A., Chung Y.C. (2013). A Coupled Biological and Photocatalysis Pretreatment System for the Removal of Crystal Violet from Wastewater. Chemosphere.

[34] Duan M., Wu J., Xiong Y., Fang S., Chen J. (2018). Characterization and Differentiation of the Adsorption Behavior of Crystal Violet and Methylene Blue at the Silica/Water Interface Using near Field Evanescent Wave. Soft Matter.

[35] Ahmad R., Hasan I. (2017). Efficient Remediation of an Aquatic Environment Contaminated by Cr (VI) and 2,4-Dinitrophenol by XG-g-Polyaniline@ZnO Nanocomposite. J. Chem. Eng. Data.

[36] Kumar N., Sinha S., Mehrotra T., Singh R., Tandon S., Thakur I.S. (2019). Biodecolorization of Azo Dye Acid Black 24 by Bacillus Pseudomycoides: Process Optimization Using Box Behnken Design Model and Toxicity Assessment. Bioresour. Technol. Rep..

[37] Bahari N.A., Isahak W.N.R.W., Masdar M.S., Ba-Abbad M.M. (2019). Optimization of the Controllable Crystal Size of Iron/Zeolite Nanocomposites Using a Box–Behnken Design and Their Catalytic Activity. Appl. Nanosci..

[38] Venkataraghavan R., Thiruchelvi R., Sharmila D. (2020). Statistical Optimization of Textile Dye Effluent Adsorption by Gracilaria Edulis Using Plackett-Burman Design and Response Surface Methodology. Heliyon.

[39] Singh R., Bhateria R. (2020). Optimization and Experimental Design of the Pb^2+^ Adsorption Process on a Nano-Fe_3_O_4_-Based Adsorbent Using the Response Surface Methodology. ACS Omega.

[40] Allouss D., Essamlali Y., Amadine O., Chakir A., Zahouily M. (2019). Response Surface Methodology for Optimization of Methylene Blue Adsorption onto Carboxymethyl Cellulose-Based Hydrogel Beads: Adsorption Kinetics, Isotherm, Thermodynamics and Reusability Studies. RSC Adv..

[41] Langmuir I. (1916). The Constitution and Fundamental Properties of Solids and Liquids. Part I. Solids. J. Am. Chem. Soc..

[42] Freundlich H.M.F. (1906). Over the Adsorption in Solution. J. Phys. Chem.

[43] Ahmad R., Hasan I. (2016). Optimization of the Adsorption of Pb (II) from Aqueous Solution onto PAB Nanocomposite Using Response Surface Methodology. Environ. Nanotechnol. Monit. Manag..

[44] Lagergren S.K. (1898). About the Theory of So-Called Adsorption of Soluble Substances. Sven. Vetenskapsakad. Handingarl.

[45] Ho Y.S., McKay G. (1999). Pseudo-Second Order Model for Sorption Processes. Process Biochem..

[46] De Franco M.A.E., De Carvalho C.B., Bonetto M.M., De Pelegrini Soares R., Féris L.A. (2018). Diclofenac Removal from Water by Adsorption Using Activated Carbon in Batch Mode and Fixed-Bed Column: Isotherms, Thermodynamic Study and Breakthrough Curves Modeling. J. Clean. Prod..

[47] Albayati T.M., Kalash K.R. (2020). Polycyclic Aromatic Hydrocarbons Adsorption from Wastewater Using Different Types of Prepared Mesoporous Materials MCM-41in Batch and Fixed Bed Column. Process Saf. Environ. Prot..

[48] Alardhi S.M., Albayati T.M., Alrubaye J.M. (2020). Adsorption of the Methyl Green Dye Pollutant from Aqueous Solution Using Mesoporous Materials MCM-41 in a Fixed-Bed Column. Heliyon.

[49] Charola S., Yadav R., Das P., Maiti S. (2018). Fixed-Bed Adsorption of Reactive Orange 84 Dye onto Activated Carbon Prepared from Empty Cotton Flower Agro-Waste. Sustain. Environ. Res..

[50] Thomas H.C. (1944). Heterogeneous Ion Exchange in a Flowing System. J. Am. Chem. Soc..

[51] Prasad A.L., Santhi T., Manonmani S. (2015). Recent Developments in Preparation of Activated Carbons by Microwave: Study of Residual Errors. Arab. J. Chem..

[52] Maruthapandi M., Kumar V.B., Luong J.H.T., Gedanken A. (2018). Kinetics, Isotherm, and Thermodynamic Studies of Methylene Blue Adsorption on Polyaniline and Polypyrrole Macro-Nanoparticles Synthesized by C-Dot-Initiated Polymerization. ACS Omega.

[53] Vijayakumar V., Khastgir D. (2018). Hybrid Composite Membranes of Chitosan/Sulfonated Polyaniline/Silica as Polymer Electrolyte Membrane for Fuel Cells. Carbohydr. Polym..

[54] Zhu X., Zhao J., Wang C. (2016). Acid and Base Dual-Controlled Cargo Molecule Release from Polyaniline Gated-Hollow Mesoporous Silica Nanoparticles. Polym. Chem..

[55] Bilal S., Akbar A., Shah A.-H.A. (2019). Highly Selective and Reproducible Electrochemical Sensing of Ascorbic Acid Through a Conductive Polymer Coated Electrode. Polymers.

[56] Karthik R., Meenakshi S. (2014). Removal of Hexavalent Chromium Ions Using Polyaniline/Silica Gel Composite. J. Water Process Eng..

[57] Scherrer P. (1918). Estimation of the Size and Internal Structure of Colloidal Particles by Means of Rontgen Rays. Nachr. Ges. Wiss. Göttingen.

[58] Sasidharan M., Mal N.K., Bhaumik A. (2007). In-Situ Polymerization of Grafted Aniline in the Channels of Mesoporous Silica SBA-15. J. Mater. Chem..

[59] Lin Y.S., Abadeer N., Hurley K.R., Haynes C.L. (2011). Ultrastable, Redispersible, Small, and Highly Organomodified Mesoporous Silica Nanotherapeutics. J. Am. Chem. Soc..

[60] Das D., Yang Y., O’Brien J.S., Breznan D., Nimesh S., Bernatchez S., Hill M., Sayari A., Vincent R., Kumarathasan P. (2014). Synthesis and Physicochemical Characterization of Mesoporous SiO_2_ Nanoparticles. J. Nanomater..

[61] Dogra P., Adolphi N.L., Wang Z., Lin Y.S., Butler K.S., Durfee P.N., Croissant J.G., Noureddine A., Coker E.N., Bearer E.L. (2018). Establishing the Effects of Mesoporous Silica Nanoparticle Properties on in Vivo Disposition Using Imaging-Based Pharmacokinetics. Nat. Commun..

[62] Zu L., Cui X., Jiang Y., Hu Z., Lian H., Liu Y., Jin Y., Li Y., Wang X. (2015). Preparation and Electrochemical Characterization of Mesoporous Polyaniline-Silica Nanocomposites as an Electrode Material for Pseudocapacitors. Materials.

[63] Lee S., Lee J., Song M.K., Ryu J.C., An B., Lee C.G., Park C., Lee S.H., Choi J.W. (2015). Effective Regeneration of an Adsorbent for the Removal of Organic Contaminants Developed Based on UV Radiation and Toxicity Evaluation. React. Funct. Polym..

[64] Casbeer E., Sharma V.K., Li X.Z. (2012). Synthesis and Photocatalytic Activity of Ferrites under Visible Light: A Review. Separation and Purification Technology.

[65] Hassaninejad-Darzi S.K., Mousavi H.Z., Ebrahimpour M. (2017). Biosorption of Acridine Orange and Auramine O Dyes onto MCM-41 Mesoporous Silica Nanoparticles Using High-Accuracy UV–Vis Partial Least Squares Regression. J. Mol. Liq..

[66] Wu Y.H., Ma Y.L., Sun Y.G., Xue K., Ma Q.L., Ma T., Ji W.X. (2020). Graded Synthesis of Highly Ordered MCM-41 and Carbon/Zeolite Composite from Coal Gasification Fine Residue for Crystal Violet Removal. J. Clean. Prod..

[67] Mahmoudi F., Amini M.M. (2020). Confined Crystallization of Microporous Metal-Organic Framework within Mesoporous Silica with Enhanced Hydrostability: Ultrafast Removal of Organic Dyes from Aqueous Solutions by MIL-68(Al)@SBA-15 Composite. J. Water Process Eng..

[68] Liang Z., Zhao Z., Sun T., Shi W., Cui F. (2017). Enhanced Adsorption of the Cationic Dyes in the Spherical CuO/Meso-Silica Nano Composite and Impact of Solution Chemistry. J. Colloid Interface Sci..

