# Supplementary material for: L-Ascorbic Acid-g-Polyaniline Mesoporous Silica Nanocomposite for Efficient Removal of Crystal Violet: A Batch and Fixed Bed Breakthrough Studies
Source: Nanomaterials (Basel). 2020 Nov 30;10(12):2402. doi: 10.3390/nano10122402 (PMC7760523; doi:10.3390/nano10122402)
Supplement: Supplementary file 1 [file nanomaterials-10-02402-s001.pdf]

nanomaterials

Supplementary Materials

L-Ascorbic Acid-g-Polyaniline Mesoporous Silica Nanocomposite for Efficient Removal of Crystal Violet: A Batch and Fixed Bed Breakthrough Studies

Imran Hasan [1], Ibtisam I. BinSharfan [2], Rais Ahmad Khan [2] and Ali Alsalme [2,*]

[1] Environmental Research Laboratory, Department of Chemistry, Chandigarh University, Gharuan, Mohali, Punjab 140301, India; imranhasan98@gmail.com

[2] Department of Chemistry, College of Science, King Saud University, Riyadh 11451, Saudi Arabia; 437202977@student.ksu.edu.sa (I.I.B.), krais@ksu.edu.sa (R.A.K.)

* Correspondence: aalsalme@ksu.edu.sa

Table S1. Variable table containing the minimum, mean and maximum values of parameters affecting adsorption reaction.

Factor	Name	Units	Type	Subtype	Minimum (−1)	Mean (0)	Maximum (+1)
A	Sonication Time	min	Numeric	Continuous	60	90	120
B	pH		Numeric	Continuous	4	5.5	7
C	CV Conc	mg L^{-1}	Numeric	Continuous	30	45	60
D	Adsorbent Dose	mg	Numeric	Continuous	20	30	40

Table S2. Design of the experiments proposed by BBD model and their response.

Standard Order	Run Order	Sonication Time (min)	pH	CV Concentration (mg L^{-1})	Adsorbent Dose (mg)	Response (mg g^{-1})
13	13	90	4	30	30	10.0196
23	23	90	4	45	40	10.4412
7	7	90	5.5	30	40	10.4559
17	17	60	5.5	30	30	10.8627
1	1	60	4	45	30	12.0000
18	18	120	5.5	30	30	12.9216
12	12	120	5.5	45	40	14.5294
24	24	90	7	45	40	15.1912
21	21	90	4	45	20	16.9706
14	14	90	7	30	30	17.1373
2	2	120	4	45	30	17.2549
26	26	90	5.5	45	30	17.4314
27	27	90	5.5	45	30	17.7255
28	28	90	5.5	45	30	17.8431
3	3	60	7	45	30	18.1176
25	25	90	5.5	45	30	18.5686
10	10	120	5.5	45	20	18.8529
11	11	60	5.5	45	40	18.8529
5	5	90	5.5	30	20	19.4412
4	4	120	7	45	30	19.4706
8	8	90	5.5	60	40	19.5588
19	19	60	5.5	60	30	20.3137
15	15	90	4	60	30	22.5294
29	29	90	5.5	45	30	23.1569
20	20	120	5.5	60	30	24.1961
16	16	90	7	60	30	27.0784
6	6	90	5.5	60	20	31.3824
9	9	60	5.5	45	20	35.6765
22	22	90	7	45	20	37.0882

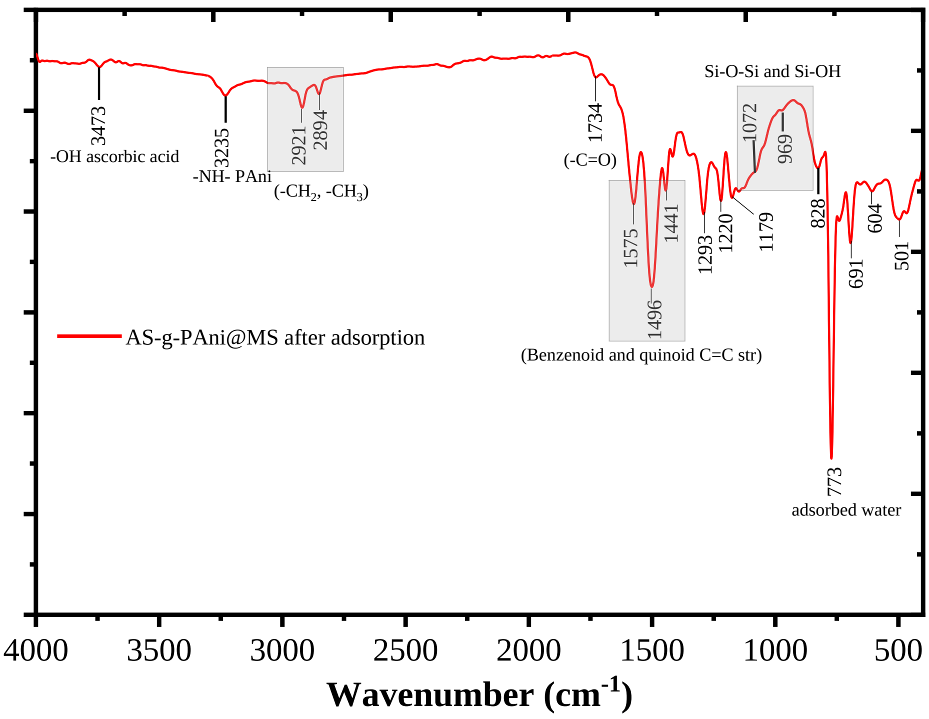

Figure S1. FTIR spectra of AS-g-PAni@MS nanocomposite after adsorption of CV dye.

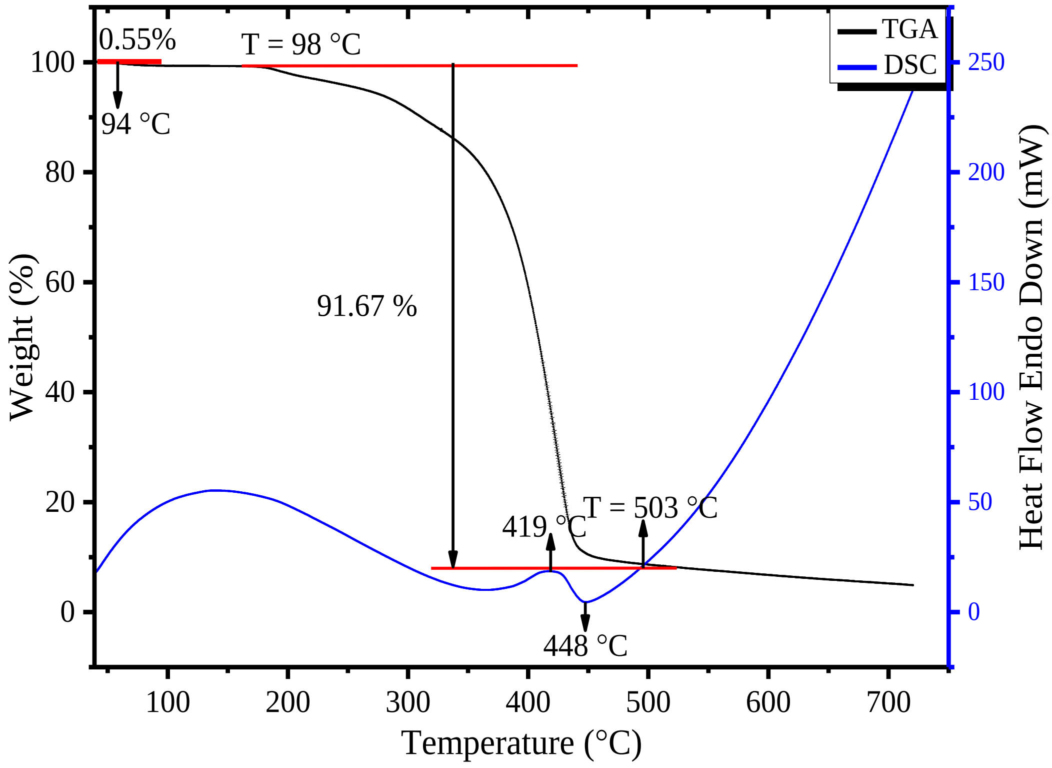

Figure S2. TGA-DSC curve to explain the thermal behaviour of AS-g-PAni@MS nanocomposite.

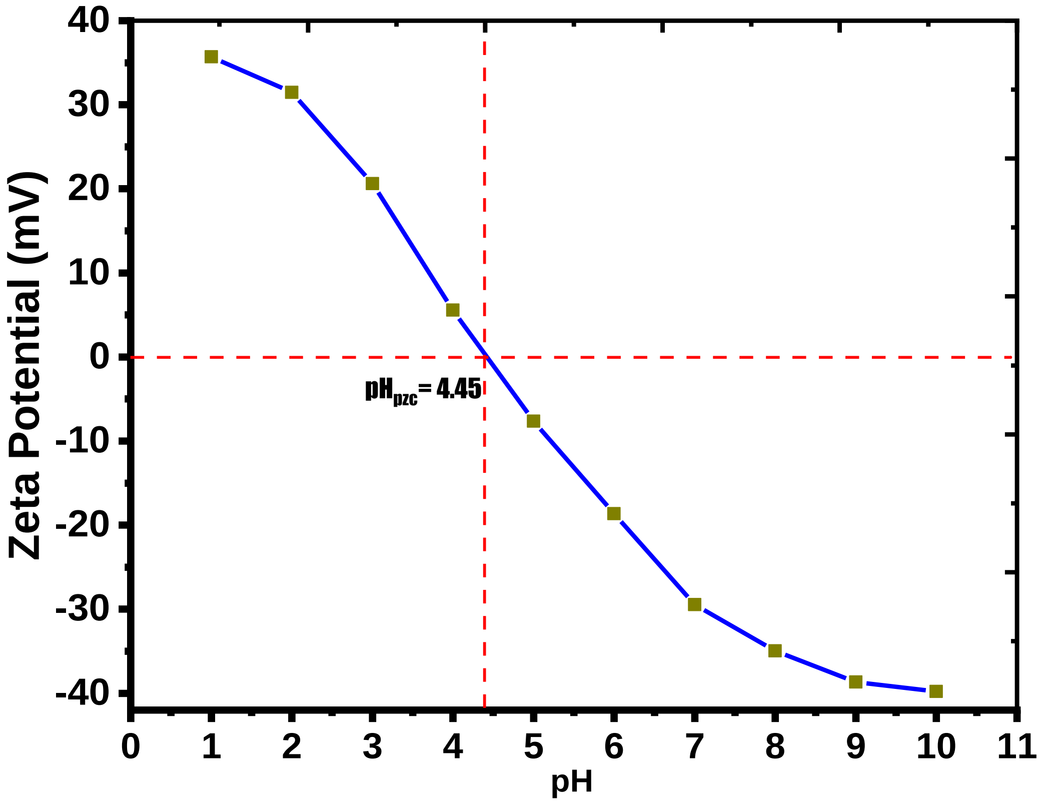

Figure S3. Zeta potential curve to obtain the point of zero charge of the nanocomposite material.

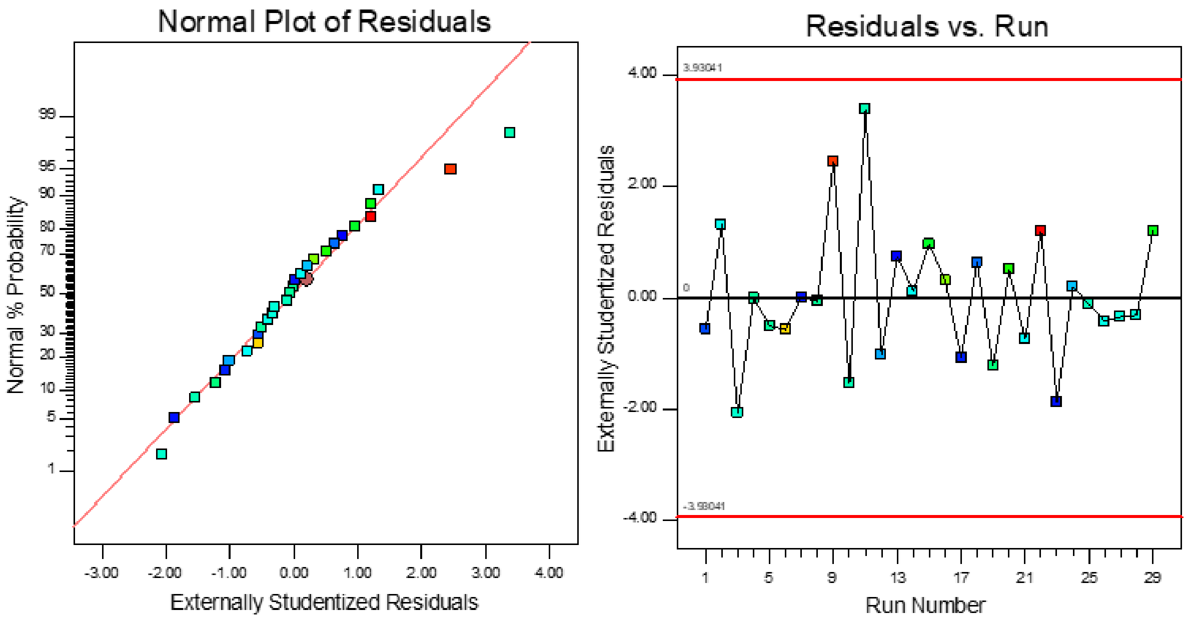

(**a**) (**b**)

Figure S4. (**a**) Normal Probability Curve (**b**) Residual vs. Run plot obtained through RSM-BBD analysis.

Table S3. Adsorption isotherm parameters obtained for the adsorption reaction of CV with AS-g-PAni@MS nanocomposite at 303–333 K obtained through nonlinear regression.

Isotherm Model	Equations	Parameters	303 K	313 K	323K	333 K
Langmuir	$qe = \frac{q_m K_L c_e}{1 + K_L c_e}$	q_m (mg g^{-1})	81.96	92.81	105.28	122.15
		K_L (L mg^{-1})	0.044	0.075	0.093	0.115
		R^2	0.99	0.97	0.99	0.97
		RMSE	0.014	0.026	0.022	0.038
Freundlich	$qe = K_F ce^{1/n}$	K_F (mg g^{-1}) (L mg^{-1})$^{1/n}$	5.42	8.07	10.12	11.72
		n	1.29	1.36	1.43	1.53
		R^2	0.98	0.96	0.98	0.96
		RMSE	0.07	0.16	0.18	0.18

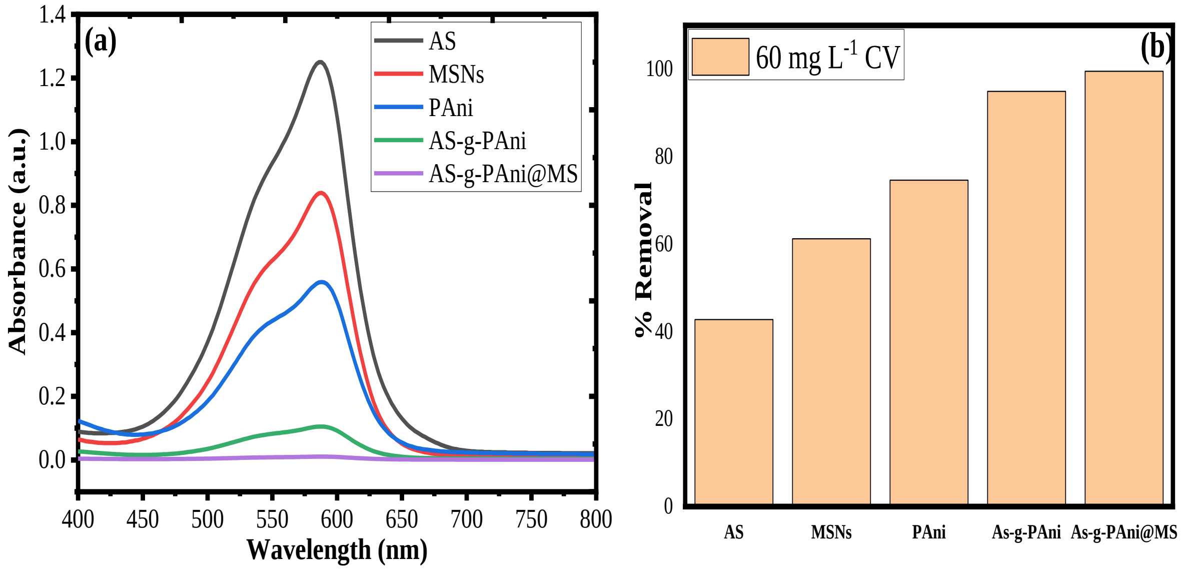

Figure S5. (**a**) UV-Vis Spectra for CV adsorption by AS-g-PAni@MS and its individual constituents (**b**) removal rate (%) for AS, MSNs, PAni, AS-g-PAni and AS-g-PAni@MS nanocomposite for 60 mg L^{-1} CV at pH 7.
